# Obesity-Induced PLPE–PTK6–FASN Feedback Loop Drives Enzalutamide Resistance by Exacerbating Lipid Metabolic Dysregulation in Castration-Resistant Prostate Cancer

**DOI:** 10.34133/research.1461

**Published:** 2026-10-15

**Authors:** Zhuo Xing, Xuewen Jiang, Banking Shi, Ruilin Liu, Ruijiang Zeng, Shangqing Ren, Zexian Ding, Pingxia Lin, Xin Yang, Jinxiang Wang, Wenjun Mao, Xin Jin

**Affiliations:** ^1^ Department of Urology, The Second Xiangya Hospital, Central South University, Changsha, Hunan, China.; ^2^ Department of Urology, Qilu Hospital, Cheeloo College of Medicine, Jinan, Shandong, China.; ^3^ Key Laboratory of Urinary Precision Diagnosis and Treatment in Universities of Shandong, Jinan, Shandong, China.; ^4^ Shandong Provincial Engineering Research Center for Precision Diagnosis and Treatment of Urological and Male Reproductive Diseases, Jinan, Shandong, China.; ^5^ Robotic Minimally Invasive Surgery Center, Sichuan Provincial People’s Hospital, School of Medicine, University of Electronic Science and Technology of China, Chengdu, Sichuan, China.; ^6^ Guilin Hospital of the Second Xiangya Hospital of Central South University, Guilin, Guangxi, China.; ^7^ Department of Urology, Pelvic Floor Disorders Center, The Seventh Affiliated Hospital, Sun Yat-sen University, Shenzhen, Guangdong, China.; ^8^ Department of Thoracic Surgery, The Affiliated Wuxi People’s Hospital of Nanjing Medical University, Wuxi People’s Hospital, Wuxi Medical Center, Nanjing Medical University, Wuxi, Jiangsu, China.; ^9^ FuRong Laboratory, Changsha, Hunan, China.

## Abstract

Obesity represents an independent risk factor for various malignancies, including prostate cancer. However, how specific lipid metabolites influence enzalutamide sensitivity in castration-resistant prostate cancer (CRPC) remains elusive. Here, we find that obesity is associated with poorer clinical outcomes during enzalutamide treatment. Lipidomic profiling reveals that 1-palmitoyl-2-linoleoyl phosphatidylethanolamine (PLPE) is markedly enriched in tumors from these individuals. Mechanistically, elevated PLPE directly binds to the SH2 domain of protein tyrosine kinase 6 (PTK6), driving its membrane translocation and functional activation. Furthermore, PLPE facilitates PTK6-fatty acid synthase (FASN) interaction, enabling PTK6 to mediate FASN phosphorylation at T976 via dual mechanisms: steric occupancy and kinase cascade amplification. This hyperactivation of FASN exacerbates lipid metabolic dysregulation, ultimately conferring enzalutamide resistance. Importantly, FASN-derived palmitic acid serves as a substrate for further PLPE synthesis, thereby reinforcing PTK6 membrane retention in a positive feedback loop. Collectively, this study identifies a PLPE–PTK6–FASN feedback loop that drives lipid metabolic reprogramming and enzalutamide resistance in CRPC with obesity, highlighting the therapeutic potential of cotargeting lipid metabolism and kinase signaling.

## Introduction

Prostate cancer (PCa) represents one of the most frequently diagnosed malignancies in males and a leading cause of cancer-associated mortality in males globally, with its incidence rising annually [1,2]. While primary PCa is typically managed through active surveillance, radical prostatectomy, or radiation therapy, metastatic PCa represents a critical stage that markedly impacts patient prognosis [3]. Androgen deprivation therapy (ADT) remains the first-line standard treatment for metastatic PCa, utilized throughout the patient’s treatment course. Despite initial therapeutic benefits, acquired resistance to ADT invariably occurs in many cases, progressing to castration-resistant prostate cancer (CRPC) [4]. The progression to CRPC is driven by the aberrant reactivation of the androgen receptor (AR) cascade [5,6]. As standard frontline interventions, novel AR pathway inhibitors such as abiraterone and enzalutamide effectively delay disease progression and enhance life quality [7,8]. However, the eventual emergence of enzalutamide resistance is inevitable [9]. Exploring the mechanisms underlying enzalutamide resistance is crucial for improving treatment outcomes for CRPC patients.

In recent decades, the global prevalence of metabolic disorders, particularly obesity, has emerged as a critical public health concern [10]. Obesity serves as an independent risk factor for various malignancies, notably encompassing breast, colorectal, and prostate carcinomas [11]. Obesity orchestrates tumor advancement through a complex network of systemic alterations, such as insulin/IGF-1 cascade dysregulation, sex steroid modulation, and adipocytokine-mediated reshaping of the immune microenvironment [12]. Wilson et al.’s study [13] highlights that obesity markedly contributes to PCa recurrence, accelerates CRPC development post-ADT, and increases PCa-specific mortality. Furthermore, PCa patients often experience weight gain following ADT [14], suggesting a potential interaction between obesity and PCa therapies. However, the impact of obesity on enzalutamide sensitivity remains elusive.

Lipidomics, through comprehensive identification, structural analysis, and quantitative assessment of the complex networks and metabolic pathways of lipids in cells or tissues, allows for the discovery of novel lipid biomarkers and targetable metabolic regulatory sites, providing key insights into disease diagnosis and treatment strategy optimization. Increasing evidence underscores the critical involvement of lipid metabolites in driving pathological progression. For instance, increased phospholipid unsaturation facilitates metastasis in triple-negative breast cancer [15], while altered ceramide metabolism has been shown to critically influence colorectal cancer progression [16]. Furthermore, specific sphingolipids promote inflammatory cascades in nonalcoholic steatohepatitis [17]. Understanding how obesity-associated lipid dysregulation impacts enzalutamide efficacy is critical for the early identification of survival benefits and the development of therapeutic targets for CRPC.

Our study aims to assess the impact of obesity on enzalutamide responsiveness in PCa, further delineating the molecular mechanisms. In this study, we found that obesity accelerates clinical progression in CRPC patients undergoing enzalutamide treatment. Through nontargeted lipidomic profiling, we identified 1-palmitoyl-2-linoleoyl phosphatidylethanolamine (PLPE) as a critical lipid metabolite enriched in obese patients that drives enzalutamide resistance. Mechanistically, elevated PLPE binds to protein tyrosine kinase 6 (PTK6) and reshapes its intracellular localization. Furthermore, PLPE strengthens the interaction between PTK6 and fatty acid synthase (FASN), thereby promoting FASN hyperphosphorylation through steric occupancy and kinase cascade amplification. The elevated phosphorylation of FASN accelerates lipid metabolism dysregulation and enzalutamide treatment resistance. The high palmitic acid generated by FASN serves as a substrate to promote PLPE synthesis, further reinforcing PTK6 membrane localization. Overall, we identified a novel PLPE–PTK6–FASN feedback loop in obesity-associated CRPC, accelerating enzalutamide resistance.

## Results

### Obesity-associated PLPE accumulation reduces enzalutamide sensitivity in PCa

Recent analyses of the ENZAMET phase III randomized trial revealed that PCPro in the circulating lipidome markedly worsened the prognosis of enzalutamide-treated metastatic hormone-sensitive PCa patients [18]. Building upon this, we hypothesized that obesity-associated lipid metabolism dysregulation might affect enzalutamide sensitivity in CRPC patients. To investigate this clinical correlation, we conducted a multicenter, retrospective cohort analysis of 137 CRPC patients treated with enzalutamide between 2022 and 2025. Then, we stratified the study population into 4 distinct subgroups according to the National Health Commission of China body mass index (BMI) criteria. Subgroup analyses showed that patients with an abnormal BMI exhibited a trend toward poorer prognosis (Fig. S1A). Notably, after excluding the confounding effects of the underweight and overweight subgroups, a distinct prognostic divergence emerged when strictly comparing the normal-weight cohort (18.5 ≤ BMI < 24 kg/m^2^, *n* = 86) with the obesity cohort (BMI ≥ 28 kg/m^2^, *n* = 29). Kaplan–Meier survival analysis revealed that obesity was significantly associated with poorer clinical outcomes and accelerated disease progression during enzalutamide treatment, as evidenced by a significant reduction in both prostate-specific antigen progression-free survival (PSA-PFS; *P* = 0.037) and radiological progression-free survival (rPFS; *P* = 0.041) (Fig. 1A). Similarly, overall survival (OS) exhibited a trend toward an adverse prognosis, although an extended follow-up period is required for definitive confirmation (*P* = 0.071) (Fig. S1B). Furthermore, after adjusting for confounding covariates, obesity-associated lipid metabolic dysregulation remained an independent risk factor driving CRPC progression (PSA-PFS: hazard ratio = 1.769, 95% confidence interval [CI] = 1.059 to 2.956, *P* = 0.0292; rPFS: hazard ratio = 1.804, 95% CI = 1.014 to 3.209, *P* = 0.0448) (Fig. 1B and Fig. S1C to F). To explore the underlying lipidomic alterations, we performed nontargeted lipidomics and liquid chromatography–tandem mass spectrometry (LC–MS/MS) quantification analysis on PCa tissue samples from CRPC patients with or without obesity (Fig. 1C to E). The results revealed that phosphatidylethanolamine (PE), phosphatidylcholine, and ceramide were the most significantly altered lipid metabolites, with the PE class exhibiting the most pronounced up-regulation (Fig. 1F and Fig. S2A). This aligns with observations in individuals with obesity, where PE concentrations increase during disease progression [19]. Consequently, we focused on PE-related lipid metabolites. Among them, PE (16:0/18:2) was not only the most significantly up-regulated metabolite in the PE class but also one of the most markedly up-regulated metabolites across the 3 lipid classes (Fig. 1G and Fig. S2B). 1-Palmitoyl-2-linoleoyl PE (PE 16:0/18:2, PLPE) is a major subtype of PE involved in maintaining membrane stability and fluidity and serves as a regulator of membrane protein localization, enzymatic activity, and signal pathways [20]. To rigorously quantify intratumoral PLPE accumulation, we performed targeted LC–MS/MS analysis on PCa specimens from both the discovery and additional validation cohorts, revealing a striking elevation of PLPE in the PCa tissues of obese patients compared to normal-weight individuals (Fig. 1H and Fig. S2C to F). Crucially, Spearman correlation analyses demonstrated that intratumoral PLPE concentrations exhibited positive associations with continuous systemic metabolic indices, specifically BMI (*R* = 0.764, *P* < 0.0001), serum triglycerides (TAGs) (*R* = 0.698, *P* < 0.0001), and serum cholesterol (*R* = 0.445, *P* = 0.0228) (Fig. 1I and Fig. S2G). Moreover, both in vitro and in vivo assays revealed that PLPE drives tumor proliferation (Fig. S2H to M). Thus, we hypothesize that elevated PLPE levels in obesity may impair enzalutamide sensitivity in PCa. Cell proliferation and apoptosis assays confirmed that PLPE promotes tumor cell proliferation and inhibits enzalutamide sensitivity (Fig. 1J to M). To further validate these findings, a high-fat diet (HFD)-driven obese mouse model was established to assess the impact on enzalutamide sensitivity and investigate the critical role of PLPE in this model (Fig. S3A to D). Consistent with our clinical observations, intratumoral PLPE levels were also significantly elevated in the HFD-fed PCa mouse models (Fig. S3E). In vivo models (cell-derived xenograft, patient-derived xenograft, and spontaneous PCa) demonstrated that high-fat feeding accelerated tumor progression and suppressed enzalutamide sensitivity. Similarly, these findings were confirmed in mouse model pretreated with PLPE (Fig. 1N to V and Fig. S3F to K). This indicated that PLPE treatment could partially simulate the obese tumor microenvironment and induce resistance to enzalutamide treatment. Collectively, our clinical observations demonstrate poorer outcomes in obese CRPC patients undergoing enzalutamide therapy. Elevated PLPE in the obese tumor microenvironment suppresses enzalutamide sensitivity by promoting tumor cell proliferation.

**Fig. 1.**
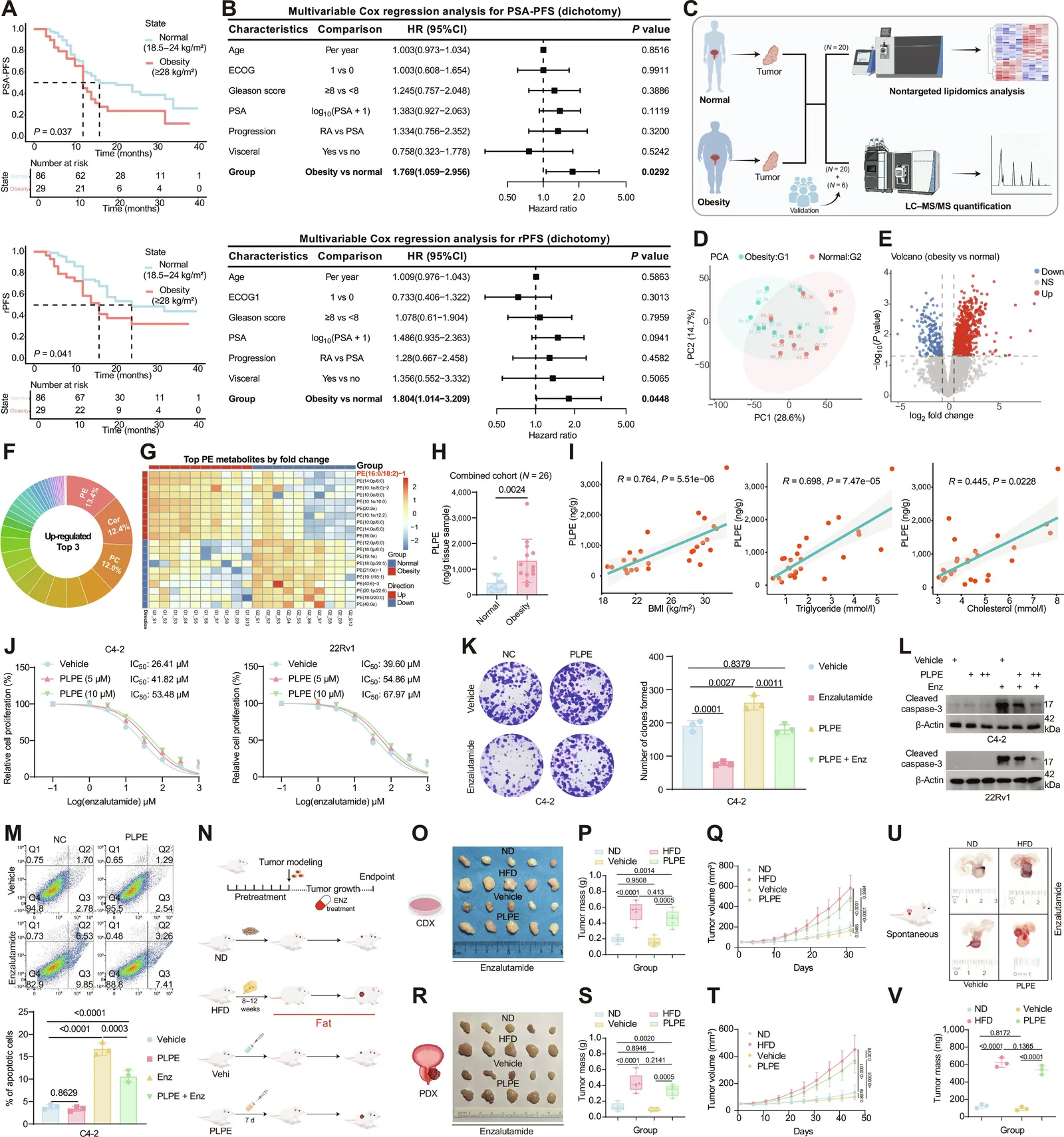
Obesity-associated 1-palmitoyl-2-linoleoyl phosphatidylethanolamine (PLPE) accumulation reduces enzalutamide sensitivity in prostate cancer (PCa). (A) Kaplan–Meier survival curves of prostate-specific antigen progression-free survival (PSA-PFS) and radiological progression-free survival (rPFS) in enzalutamide-treated castration-resistant prostate cancer (CRPC) patients, comparing the normal weight (18.5 ≤ body mass index [BMI] < 24 kg/m^2^, *n* = 86) and obesity (BMI ≥ 28 kg/m^2^, *n* = 29) subgroups. (B) Multivariable Cox regression analyses for PSA-PFS and rPFS evaluating the independent prognostic value of the dichotomous BMI subgroups (obesity vs normal weight). (C) Flow diagram outlining the collection of PCa tumor samples from CRPC patients with or without obesity for nontargeted lipidomics and liquid chromatography–tandem mass spectrometry (LC–MS/MS) quantification analysis. (D to G) Principal component analysis (PCA) (D), volcano plot (E), donut chart (F), and heatmap of the top differentially expressed phosphatidylethanolamine (PE) metabolites (G) from the nontargeted lipidomics analysis. (H) Absolute quantification of intratumoral PLPE levels in CRPC tissue samples from the combined cohort (normal vs obesity) using targeted LC–MS/MS (*n* = 13 per group, Student *t* test). (I) Spearman correlation analyses between intratumoral PLPE concentrations and continuous clinical metabolic indices, including BMI, serum triglycerides, and serum cholesterol. (J to M) PCa cells were treated with the vehicle, PLPE (5/10 μM), or enzalutamide. Subsequently, the collected cells were subjected to Cell Counting Kit-8 (CCK-8) (J), colony formation (K), Western blotting (L), and annexin V/propidium iodide (PI) flow cytometry (M). (N) Schematic diagram of the in vivo animal experimental design. Mice were randomized to receive a normal diet group (ND, containing 10% kcal fat, 70% kcal carbohydrates, and 20% kcal protein) or a high-fat diet group (HFD, containing 60% kcal fat, 20% kcal carbohydrates, and 20% kcal protein) for 8 to 12 weeks to establish normal-weight and obese mouse models. Another batch of mice was randomly assigned to receive vehicle or PLPE pretreatment (1 mg/kg, intraperitoneal) for 7 d to construct normal-weight and high-PLPE mouse models. Subsequently, in vivo tumor formation experiments were performed on mice in each group; upon the tumors attaining an approximate volume of 50 mm^3^, mice received daily (q.d.) oral gavage of enzalutamide (10 mg/kg). The original modeling intervention conditions were maintained throughout the experiment. (O to Q) A subcutaneous cell-line-derived xenograft (CDX) model of PCa was established utilizing C4-2 cells. Representative tumors images (O), tumor mass (P), and tumor growth curves (Q) (*n* = 5 per group, one-way analysis of variance [ANOVA] with Tukey’s test). (R to T) A patient-derived xenograft (PDX) model of PCa was established utilizing clinical tumor specimens. Representative tumors images (R), tumor mass (S), and tumor growth curves (T) (*n* = 5 per group, one-way ANOVA with Tukey’s test). (U and V) A spontaneous PCa tumor model was established by plasmid injection. Representative tumors images (U) and tumor mass (V) (*n* = 3 per group, Student *t* test).

### PLPE enhances the membrane localization of PTK6 and suppresses enzalutamide sensitivity in PCa through PTK6

To unravel the molecular underpinnings of PLPE-driven enzalutamide resistance, we synthesized biotin-conjugated PLPE and performed protein MS to identify specific binding targets (Fig. 2A and Fig. S4A). Combined analyses revealed that PTK6 was up-regulated in PCa tissues and enzalutamide-resistant PCa cells and possessed the capability to bind PLPE (Fig. 2B and C and Fig. S4B). Consistently, metabolomic profiling of PTK6-enriched complexes verified the direct interaction between PTK6 and PLPE (Fig. 2D). PTK6, an intracellular tyrosine kinase, is commonly overexpressed in numerous malignancies and associated with poor prognosis [21]. Bioinformatic evaluations further demonstrated that PTK6 up-regulation in PCa is significantly associated with advanced disease stages and unfavorable clinical prognoses (Fig. S4C to J). Moreover, CRISPR–Cas9 kinase library screen by Palit et al. identified PTK6 among the top 10% of genes dictating enzalutamide sensitivity in PCa cells (Fig. 2E). In agreement, PTK6 expression was markedly higher in enzalutamide-resistant cells than in parental PCa cells (Fig. 2F). Integrated analysis revealed that AR was the most frequently reported transcription factor of PTK6 (Fig. S5A). As previously noted, the restoration of AR signaling serves as a major driver of CRPC and contributes to enzalutamide resistance [5]. However, unlike the suppression of classical androgen response element-dependent target genes (e.g., KLK3 and TMPRSS2) in therapy-resistant CRPC [22], PTK6 may be transcriptionally activated by the AR–TET2–CXXC5 complex through a nonclassical pathway (Fig. S5B to H) [23]. Functional assays on cell proliferation and apoptosis demonstrated that PTK6 acted as a critical regulator of enzalutamide therapeutic sensitivity in PCa. Notably, PTK6-driven enzalutamide resistance was not entirely reliant on its kinase activity. Ectopic overexpression of the K219M mutant, which abolishes kinase activity [21], partially attenuated the antitumor efficacy of enzalutamide (Fig. 2G and Fig. S6A to J).

**Fig. 2.**
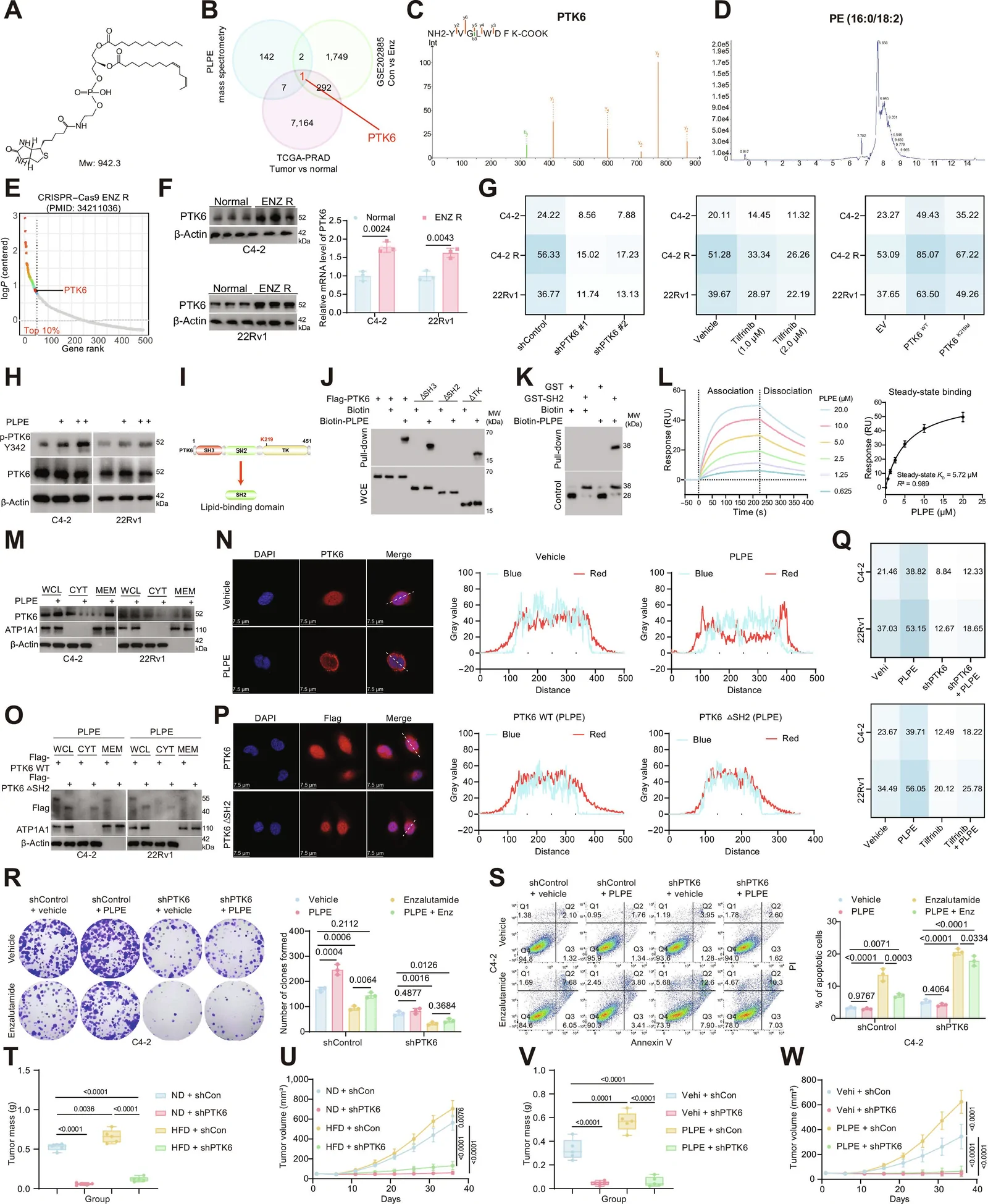
1-Palmitoyl-2-linoleoyl phosphatidylethanolamine (PLPE) enhances the membrane localization of protein tyrosine kinase 6 (PTK6) and suppresses enzalutamide sensitivity in prostate cancer (PCa) through PTK6. (A) Schematic diagram of PLPE linked by biotin. (B) Venn diagram of differentially up-regulated genes in The Cancer Genome Atlas Prostate Adenocarcinoma dataset (TCGA-PRAD), enzalutamide-resistant CRPC cells (GSE202885), and PLPE protein mass spectrometry. (C) PTK6 peptide sequences identified by PLPE mass spectrometry. (D) The lipid molecules information of phosphatidylethanolamine (PE) (16:0/18:2) in the mass spectrometry of PTK6. (E) Gene ranking plot from CRISPR–Cas9 screen of enzalutamide-resistant genes based on kinome-centered profiling. (F) The protein and messenger RNA (mRNA) expression levels of PTK6 in parental and enzalutamide-resistant PCa cells were evaluated using Western blotting and reverse transcription quantitative polymerase chain reaction (RT-qPCR) (*n* = 3, Student *t* test). (G) PCa cells, pretreated with the indicated plasmids or compounds, were further cultured with varying doses of enzalutamide for 72 h. The enzalutamide IC_50_ was then quantified utilizing the Cell Counting Kit-8 (CCK-8) assay. (H) PCa cells were treated with PLPE for 48 h, followed by Western blotting analysis. (I) Schematic diagram of the PTK6 structure. (J) Flag-tagged wild-type PTK6 and its truncation mutants were transfected into 293T cells. Following the coincubation of whole-cell extracts (WCEs) with a biotinylated PLPE probe or unconjugated biotin (negative control), immunoblotting was performed using an anti-Flag antibody. Upper: Flag-PTK6 detected in pull-down samples. Lower: WCE input control. (K) Extraction of the purified PTK6 SH2 protein with the glutathione *S*-transferase (GST) tag. The purified protein was incubated with biotinylated PLPE or biotin and then precipitated using streptavidin magnetic beads, and immunoblotting was performed with anti-GST antibody. (L) Surface plasmon resonance (SPR) analysis evaluating the direct binding affinity between PLPE and the PTK6 SH2 domain. Steady-state binding curve plotting the equilibrium response (RU) versus PLPE concentration. The equilibrium dissociation constant (*K*_D_) was determined using a steady-state affinity nonlinear regression model (*n* = 3). (M and N) PCa cells were treated with the PLPE for 48 h. Subcellular isolates were separated and evaluated via Western blotting (M) and immunofluorescence assay (N). WCL, whole-cell lysate; CYT, cytoplasm; MEM, biological membrane, which includes the plasma membrane and organelle membranes. (O and P) Following transfection with the indicated plasmids for 72 h, the PCa cells were subjected to subcellular fraction extraction. Protein distribution was then confirmed by Western blotting (O) and immunofluorescence assays (P). (Q to S) PCa cells, treated with the indicated plasmids or chemical agents, were harvested for CCK-8 assay (Q), colony formation assay (R), and annexin V/propidium iodide (PI) flow cytometry (S) (*n* = 3, one-way analysis of variance [ANOVA] with Tukey’s test). (T and U) Control and PTK6-silenced C4-2 cells were subcutaneously implanted into standard-diet and obesity-model mice, with the specific feeding protocols maintained until the endpoint. Upon the tumors attaining an approximate volume of 50 mm^3^, mice were treated with enzalutamide (10 mg/kg, q.d.) via oral gavage. Tumor mass (T) and tumor growth curve (U) (*n* = 5 per group, one-way ANOVA with Tukey’s test). (V and W) Control and PTK6-silenced C4-2 cells were subcutaneously implanted into vehicle- or PLPE-pretreated mice, with the designated treatment regimens maintained until the endpoint. Upon the tumors attaining an approximate volume of 50 mm^3^, mice were treated with vehicle or enzalutamide (10 mg/kg, q.d.) via oral gavage. Tumor mass (V) and tumor growth curve (W) (*n* = 5 per group, one-way ANOVA with Tukey’s test).

We next investigated how PLPE modulates PTK6. Our findings indicated that PLPE treatment had no effect on total PTK6 expression but markedly increased PTK6 Y342 phosphorylation and the subsequent activation of multiple downstream substrates (Fig. 2H and Fig. S7A and B). Wozniak et al. [24] demonstrated that PTEN deficiency fuels PCa development by promoting PTK6 Y342 phosphorylation. Because PTK6 lacks canonical membrane or nuclear targeting motifs, its subcellular localization is highly dynamic. Zheng et al. [25] highlighted that membrane localization is essential for PTK6 activation, and the addition of a palmitoylation/myristoylation motif (Palm) to its N-terminus markedly expanded the reservoir of active PTK6 in PCa cells. Structural evaluations revealed that membrane localization is crucial for PTK6 function. Although the scaffold function provided by the K219M mutant confers a basal level of resistance, the intrinsic kinase activity of PTK6 is indispensable for driving the maximal resistant phenotype (Fig. S7C and D). Notably, the SH2 domain of PTK6 contains a unique lipid-binding pocket on its surface, and protein–lipid interactions are indispensable for PTK6 membrane targeting and functional signaling [26]. Further, using a series of PTK6 truncation mutants, we confirmed that PLPE specifically binds to the SH2 domain of PTK6, whereas the other common lipid components did not show marked binding (Fig. 2I to K and Fig. S7E and F). To further quantify this binding, surface plasmon resonance assays confirmed a direct and specific interaction between the PTK6 SH2 domain and PLPE, revealing a moderate binding affinity in the micromolar range (*K*_D_ = 5.72 μM) (Fig. 2L). Consistently, our results showed that PLPE enhanced PTK6 biological membrane (plasma/organelle) localization, whereas deletion of the SH2 domain abrogated this effect (Fig. 2M to P). We next sought to determine whether PTK6 is the key mediator through which PLPE regulates enzalutamide sensitivity. Cell proliferation and apoptosis assays revealed that PTK6 knockdown or pharmacological inhibition restored enzalutamide sensitivity and partially reversed PLPE-induced resistance (Fig. 2Q to S and Fig. S8A to D). Similarly, PTK6 knockdown suppressed tumor growth and enhanced enzalutamide responsiveness in both obesity and PLPE-pretreated xenograft models (Fig. 2T to W and Fig. S8E and F). Collectively, these data demonstrate that PLPE binds to the SH2 domain of PTK6 to promote its membrane localization and expand its active kinase pool, thereby reducing enzalutamide sensitivity in PCa.

### The PLPE–PTK6 complex modulates enzalutamide sensitivity via FASN

To elucidate the downstream effectors through which the PLPE–PTK6 complex dictates enzalutamide sensitivity, we performed transcriptomic profiling on cells treated with the PTK6 inhibitor (tilfrinib). This analysis revealed a significant enrichment of altered lipid-metabolism-related pathways (Fig. 3A and B). From the integrative analysis of PLPE/PTK6-associated protein MS, PTK6 phosphoproteomics, and lipid-metabolism-related gene sets, we identified that FASN might be a potential downstream target of the PLPE–PTK6 complex (Fig. 3C). FASN serves as a fundamental metabolic hub, driving fatty acid production to sustain critical cellular processes such as membrane biogenesis, energy deposition, signaling cascades, and protein lipidation [27]. Aberrant de novo fatty acid biosynthesis serves as a core metabolic feature in CRPC, fueling enzalutamide resistance. Combining FASN inhibitors with enzalutamide markedly augmented the antitumor efficacy in CRPC [28]. Our results show that knockdown of FASN in PCa cells reversed the inhibitory effects of PLPE or PTK6 on enzalutamide sensitivity (Fig. 3D to J and Fig. S9A to D). Moreover, PLPE–PTK6 complex formation further compromised the antitumor effects of enzalutamide (Fig. S9E to G). This finding was further confirmed in xenograft mouse models (Fig. S9H to J). Given its pivotal role in lipid metabolism, FASN represents a compelling therapeutic target, with several FASN inhibitors, such as TVB2640 and TVB3664, currently undergoing clinical trials and showing promising clinical results in the treatment of metabolic diseases and cancer [29]. In concordance with previous studies, our findings underscore that combined use of FASN inhibitors partially reversed enzalutamide resistance in CRPC cells (Fig. S10A and B). However, we observed that exogenous PLPE supplementation or PTK6 overexpression both attenuated the antitumor efficacy of FASN inhibitor combined with enzalutamide (Fig. 3K and Fig. S10C to F). Furthermore, this inhibitory effect was markedly amplified upon PLPE–PTK6 complex formation (Fig. 3L to Q and Fig. S10G). Similarly, in PLPE–PTK6-complex-pretreated animal models, the antitumor effect of combining FASN inhibitors with enzalutamide was suppressed (Fig. 3R to U). Notably, triple-agent combination treatment incorporating the PTK6 inhibitor partially reversed this therapeutic resistance phenotype, although its associated pharmacological toxicity warrants further evaluation (Fig. S11A to D). In conclusion, our results indicate that FASN was a key molecule through which the PLPE–PTK6 complex regulated enzalutamide therapeutic sensitivity. FASN inhibition partially reversed enzalutamide resistance in CRPC cells. However, the PLPE–PTK6 complex suppressed the efficacy of FASN inhibitors, thereby attenuating the antitumor effect of combined with enzalutamide.

**Fig. 3.**
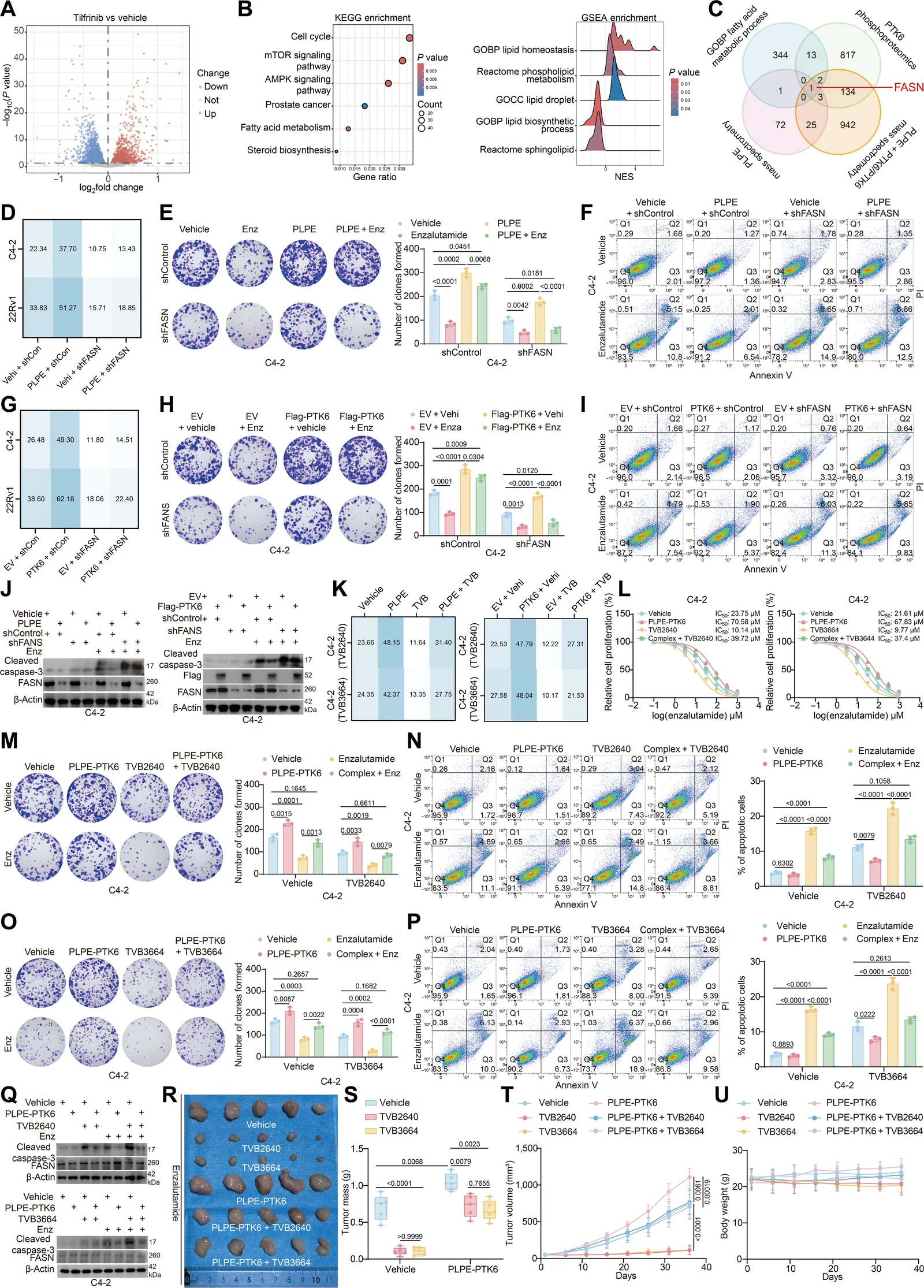
The 1-palmitoyl-2-linoleoyl phosphatidylethanolamine (PLPE)-protein tyrosine kinase 6 (PTK6) complex modulates enzalutamide sensitivity via fatty acid synthase (FASN). (A and B) Analysis of differentially expressed genes (A), and subsequent Kyoto Encyclopedia of Genes and Genomes (KEGG)/gene set enrichment analysis (GSEA) functional annotations (B) in 22Rv1 cells with indicated treatment. (C) Venn diagram of the PLPE mass spectrometry of proteins, differentially up-regulated proteins in PTK6 mass spectrometry, PTK6 phosphorylation proteomics, and fatty acid metabolic process gene set. (D to J) Prostate cancer (PCa) cells, treated with the indicated plasmids or PLPE (10 μM), were subjected to IC_50_ determination (D and G), colony formation assay (E and H), annexin V/propidium iodide (PI) flow cytometry (F and I), and Western blot (J) (*n* = 3, one-way analysis of variance [ANOVA] with Tukey’s test). (K and L) C4-2 cells, preconditioned with either PTK6 plasmids or 10 μM PLPE, were further cultured with varying doses of enzalutamide for 72 h. The IC_50_ of enzalutamide was then quantified with the Cell Counting Kit-8 (CCK-8) assay. (M to Q) PTK6-stably transfected C4-2 cells were exposed to PLPE for 48 h to generate mixed-treatment cells. Both control and experimental cells were then incubated with TVB2640 (0.2 μM) or TVB3664 (50 nM) for 48 h. Cells were harvested to colony formation assay (M and O), annexin V/PI flow cytometry (N and P), and Western blot analysis (Q) (*n* = 3, one-way ANOVA with Tukey’s test). (R to U) Subcutaneous xenografts were established using control C4-2 cells in vehicle-pretreated mice or PTK6-overexpressing C4-2 cells in PLPE-pretreated mice (PLPE–PTK6 group). The respective vehicle or PLPE treatments were maintained throughout the experiment. When tumors reached approximately 50 mm^3^, mice in each condition were randomized to receive enzalutamide (10 mg/kg) combined with vehicle, TVB2640 (5 mg/kg), or TVB3664 (3 mg/kg). Enzalutamide and the FASN inhibitors were administered daily by oral gavage. Representative tumor images (R), tumor mass (S), tumor growth curves (T), and body weight curves (U) are shown (*n* = 5 mice per group, one-way ANOVA with Tukey’s test).

### PLPE enhances the interaction between PTK6 and FASN

Previous MS screening of PTK6 initially indicated that PLPE treatment enhanced the interaction between PTK6 and FASN (Fig. 4A). PTK6 exhibited basal binding affinity toward FASN, while the intrinsic interaction was relatively weak (Fig. 4B and C). Reanalyzing the MS of biotin-labeled PLPE, we found that PLPE could directly bind to FASN (Fig. 4D). Based on previous studies and UniProt database annotations, we subdivided FASN into 2 structural domains: the N-terminal region (amino acids 1 to 1,108) and the C-terminal region (amino acids 1,109 to 2,511) [30]. Domain interaction assays showed that PLPE predominantly binds to the C-terminal region of FASN (Fig. 4E to G). Molecular docking analysis predicted that PLPE directly interacts with FASN, and the addition of PLPE markedly enhanced the binding affinity between PTK6 and FASN (Fig. 4H and I). Consistently, coimmunoprecipitation (Co-IP) assays validated this regulatory effect (Fig. 4J). Immunofluorescence colocalization assays also verified that PLPE treatment markedly promoted the intracellular colocalization of PTK6 and FASN (Fig. 4K). Collectively, PLPE markedly augments the binding affinity of PTK6 for FASN (Fig. 4L).

**Fig. 4.**
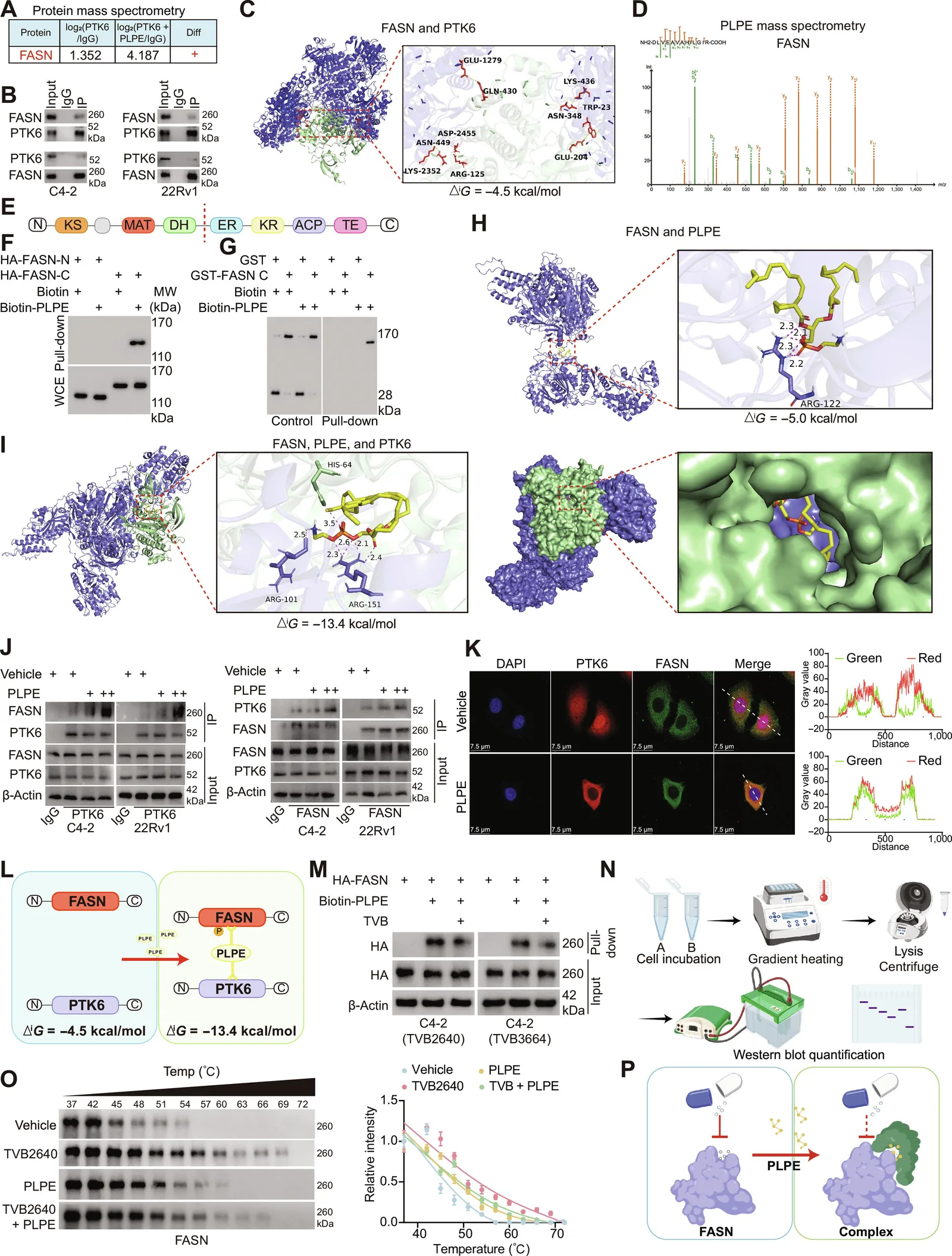
1-Palmitoyl-2-linoleoyl phosphatidylethanolamine (PLPE) enhances the interaction between protein tyrosine kinase 6 (PTK6) and fatty acid synthase (FASN). (A) Following treatment with or without PLPE for 48 h, C4-2 cells were collected for PTK6-targeted immunoprecipitation and subsequent mass spectrometric identification. (B) Coimmunoprecipitation (Co-IP) analyses of prostate cancer (PCa) cell lysates were conducted using the indicated antibodies. (C) The binding energy between PTK6 and FASN proteins was predicted using AlphaFold 3 and PDBePISA, and the interaction mode was visualized by the PyMOL software. (D) Peptide profiles of FASN identified by PLPE mass spectrometry. (E) Schematic diagram of the FASN structure and the peptide information of FASN. (F) Hemagglutinin (HA)-tagged FASN truncation mutants were transfected into 293T cells. Whole-cell lysates were exposed to biotin-labeled PLPE or biotin. Streptavidin beads were used for pull-down, and immunoblotting was performed with anti-HA antibody thereafter. (G) Extraction of the purified FASN C-terminal domain protein with the glutathione *S*-transferase (GST) tag. The purified protein was incubated with biotinylated PLPE or biotin and then precipitated using streptavidin magnetic beads, and immunoblotting was performed with anti-GST antibody. (H) The binding energy between PLPE and FASN proteins was predicted using AutoDock Vina 1.2.6, and the interaction mode was visualized by the PyMOL software. (I) The PLPE–PTK6–FASN complex was docked using the GRAMM system, and the binding energy of the complex was calculated by PDBePISA. Subsequently, the interaction interface and spatial conformation of the PLPE–PTK6–FASN complex were analyzed and visualized in the PyMOL software. (J) Following exposure to a PLPE concentration gradient for 48 h, C4-2 cells were processed for Co-IP assays. (K) Following exposure to PLPE for 48 h, C4-2 cells were processed for immunofluorescence staining. (L) Schematic diagram of PLPE promoting the interaction between PTK6 and FASN. (M) HA-tagged FASN was delivered into C4-2 cells. Cell lysates were coincubated with biotin-labeled PLPE in the presence or absence of FASN inhibitors (TVB2640 or TVB3664). Streptavidin beads were used for pull-down, and immunoblotting was performed with anti-HA antibody thereafter. (N) Schematic of the intact-cell cellular thermal shift assay (CETSA) workflow. Following compound treatment, intact cells were heated at the indicated temperatures, cooled, and lysed. Soluble FASN remaining after centrifugation was detected by immunoblotting. (O) Representative FASN immunoblots and corresponding thermal stability profiles from cells treated with vehicle, TVB2640, PLPE, or their combination. Band intensities were quantified using ImageJ. (P) Schematic diagram: PLPE enhanced the binding ability between FASN and PTK6 proteins, disrupting the binding of FASN inhibitors, thereby interfering with the targeting efficiency of FASN inhibitors.

Furthermore, we preliminarily explored how PLPE attenuated the antitumor effect of FASN inhibitors. The results showed that FASN inhibitors markedly disrupted the interaction between PLPE and FASN (Fig. 4M). Intact-cell cellular thermal shift assay showed that TVB2640 increased the thermal stability of FASN, whereas PLPE alone produced a modest stabilizing effect. Cotreatment with PLPE attenuated the TVB2640-induced thermal stabilization of FASN (Fig. 4N and O). Together with the interaction data, these findings support a model in which PLPE-enhanced PTK6–FASN association may modulate the response of FASN to TVB2640. Moreover, PLPE treatment also reduced the sensitivity of PCa cells to FASN inhibitor therapy (Fig. S12A and B). Together, these findings support a model in which PLPE binding to FASN promotes its association with PTK6, potentially altering FASN responsiveness to its inhibitors and attenuating their antitumor effects (Fig. 4P).

### The PLPE–PTK6 complex enhances FASN activity by promoting T976 phosphorylation

To dissect how PTK6 regulates FASN, we performed phosphoproteomic profiling after altering PTK6 expression. The profiles revealed that PTK6 modulates the phosphorylation status of FASN at the T976 site (Fig. 5A). Jin et al. [31] demonstrated that phosphorylated FASN enhances its activity and drives intracellular lipid accumulation. Given that PTK6 is a tyrosine kinase, it failed to directly mediate the phosphorylation-driven activation of FASN at the threonine-976 residue (Fig. 5B). However, phosphorylation assays revealed that PTK6 increased FASN phosphorylation at T976, and this effect was not fully dependent on its kinase activity (Fig. 5C and D). Consistently, PLPE treatment also elevated FASN phosphorylation levels in a PTK6-dependent manner (Fig. 5E and F). Further, we constructed FASN plasmids harboring phosphomimetic and phospho-defective mutations. We found that phosphorylation at FASN T976 did not affect its protein stability or subcellular localization, but significantly promoted its homodimerization (Fig. S13A to C). Extensive evidence has demonstrated that FASN homodimerization is essential for its full enzymatic activity [32]. Further results have confirmed that enhanced phosphorylation of FASN at T976 markedly promoted the intracellular synthesis of TAG and palmitic acid (Fig. 5G and H). Functional assays validated that T976 phosphorylation of FASN significantly reduced enzalutamide therapeutic sensitivity (Fig. 5I to K). Similarly, PLPE/PTK6 enhanced FASN activity, promoting TAG and palmitic acid generation (Fig. 5L). FASN knockdown effectively reversed the lipid metabolism disorders mediated by PLPE or PTK6 (Fig. 5M and Fig. S14A and B). Furthermore, PLPE or PTK6 treatment attenuated the inhibitory effect of FASN inhibitors on lipid synthesis (Fig. 5N and Fig. S14C to G), which was further validated by Oil Red O staining assays (Fig. 5O to Q). Since PLPE is a PE species containing palmitoyl and linoleoyl acyl chains, we reasoned that the elevated intracellular palmitic acid—a 16-carbon saturated fatty acid generated by hyperactivated FASN—acts as a substrate to further increase PLPE content. MS analysis confirmed that exogenous palmitic acid supplementation increased intracellular PLPE content (Fig. 5R). Similarly, elevated FASN phosphorylation levels significantly increased intracellular PLPE content (Fig. 5S), whereas inhibition of FASN and PTK6 markedly reduced intracellular PLPE content (Fig. 5T to V and Fig. S14H and I). These results indicate that this PLPE–PTK6–FASN feedback loop drives lipid metabolic reprogramming and promotes the acquisition of enzalutamide resistance in CRPC (Fig. 5W).

**Fig. 5.**
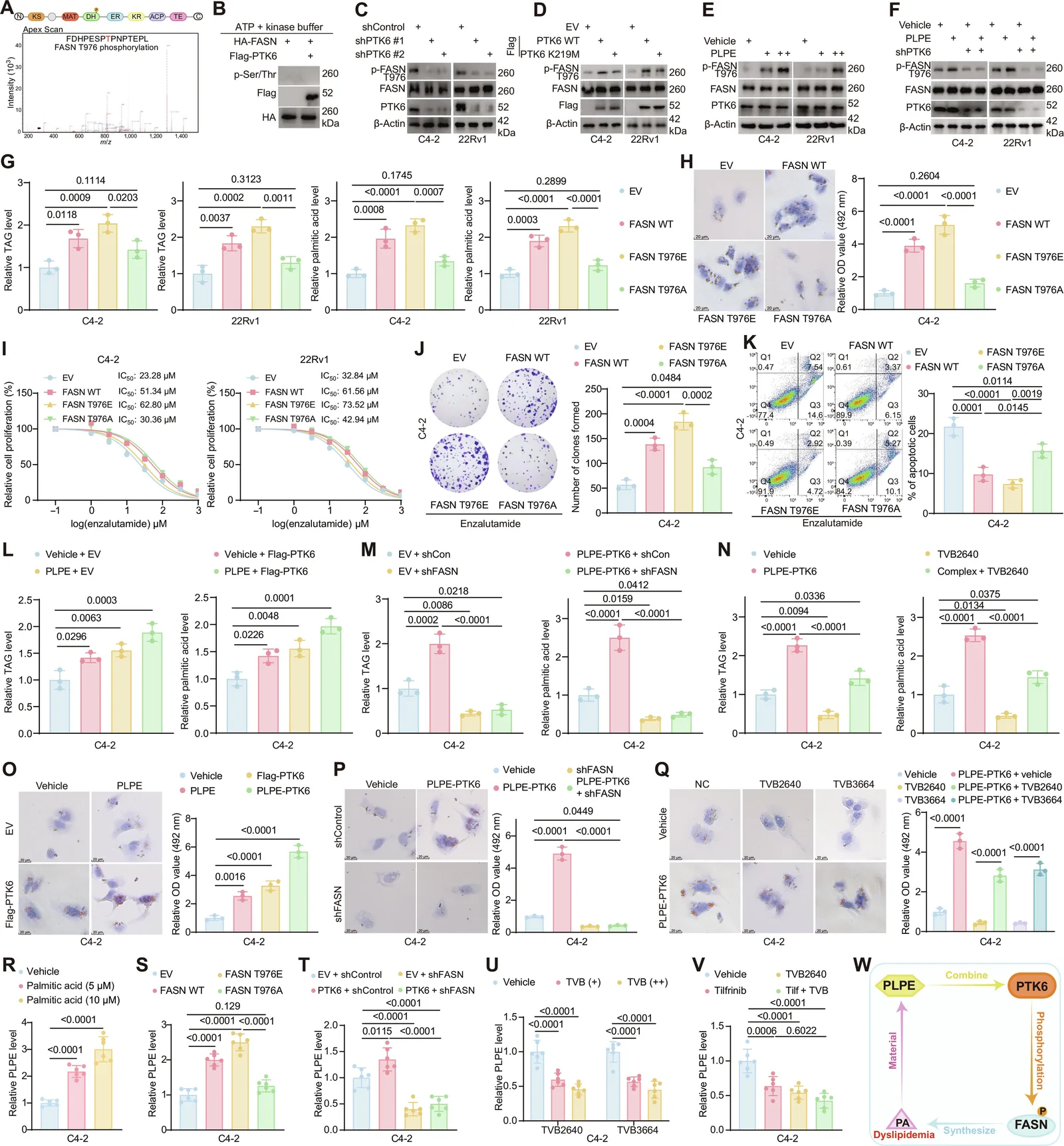
The 1-palmitoyl-2-linoleoyl phosphatidylethanolamine (PLPE)-protein tyrosine kinase 6 (PTK6) complex enhances fatty acid synthase (FASN) activity by promoting T976 phosphorylation. (A) The peptide information of FASN T976 in the phosphorylation proteomics of PTK6. (B) A purified FASN substrate and PTK6 kinase were incubated in kinase buffer containing adenosine triphosphate (ATP) for in vitro kinase reactions. Reaction products were analyzed by Western blot for the phosphorylation signal. (C to F) Following treatment with indicated plasmid or pharmacological for 72 h, prostate cancer (PCa) cells were harvested for Western blotting. (G and H) PCa cells expressing the specified plasmids were subjected to triglyceride (TAG) or palmitic acid quantification (G) and Oil Red O staining (H) (*n* = 3, one-way analysis of variance [ANOVA] with Tukey’s test). (I to K) PCa cells expressing the specified plasmids were subjected to determination of IC_50_ values (I), colony formation assay (J), and annexin V/propidium iodide (PI) flow cytometry (K) (*n* = 3, one-way ANOVA with Tukey’s test). (L to Q) Following treatment with the specified plasmids (Flag-PTK6; shFASN) or chemical (PLPE: 10 μM; TVB2640: 0.2 μM; TVB3664: 50 nM) for 72 h, PCa cells were collected for TAG or palmitic acid quantification (L to N) and Oil Red O staining (O to Q) (*n* = 3, one-way ANOVA with Tukey’s test). (R to V) Following transfection with the indicated plasmids or exposure to specific pharmacological agents for 72 h, C4-2 cells were harvested for the quantification of endogenous PLPE levels (*n* = 6, one-way ANOVA with Tukey’s test). (W) Schematic diagram of the PLPE–PTK6–FASN loop. Elevated PLPE binds to PTK6, alters its subcellular localization, and enhances the interaction between PTK6 and FASN. PTK6 promotes FASN function by mediating its phosphorylation, thereby increasing intracellular palmitic acid levels. The accumulated palmitic acid serves as a substrate to facilitate PLPE synthesis and strengthen the membrane localization of PTK6, ultimately forming a positive regulatory feedback loop.

### The PLPE–PTK6 complex drives FASN T976 phosphorylation via a kinase cascade and steric hindrance

Reviewing previous sequencing profiles revealed that suppressing PTK6 markedly perturbed kinase networks (Fig. 3B). To elucidate the exact mechanisms underlying PTK6-mediated FASN T976 phosphorylation, we integrated PTK6 MS, PTK6 phosphoproteomics, and FASN-interacting proteins (BioGRID database). Protein kinase C-zeta (PRKCZ) and cyclin-dependent kinase 2 (CDK2) were identified as potential serine/threonine kinases mediating FASN phosphorylation (Fig. 6A). Given its predominant nuclear localization, CDK2 exhibits a distinct subcellular pattern from FASN, leading us to prioritize PRKCZ for subsequent functional validation. PRKCZ has been widely reported to play a pivotal role in mediating the malignant phenotypes of PCa [33]. Endogenous Co-IP assays confirmed physical interaction among PTK6, PRKCZ, and FASN (Fig. 6B). Furthermore, our phosphoproteomics data revealed that PTK6 mediates the tyrosine phosphorylation of PRKCZ at the Y417 residue (Fig. 6C). In vitro kinase assays verified that PTK6 directly phosphorylates PRKCZ (Fig. 6D). Subsequent experiments demonstrated that PTK6 dynamically regulates PRKCZ Y417 phosphorylation in a kinase-dependent manner, and PLPE stimulation further enhances PRKCZ phosphorylation via PTK6 (Fig. 6E to H). Moving downstream, we confirmed that PRKCZ actively phosphorylates FASN at T976 in a kinase-dependent manner, and crucially, PTK6-induced FASN phosphorylation is strictly dependent on the presence of PRKCZ (Fig. 6I to L). Together, these results indicate that PTK6 activates FASN via a PRKCZ-mediated phosphorylation cascade.

**Fig. 6.**
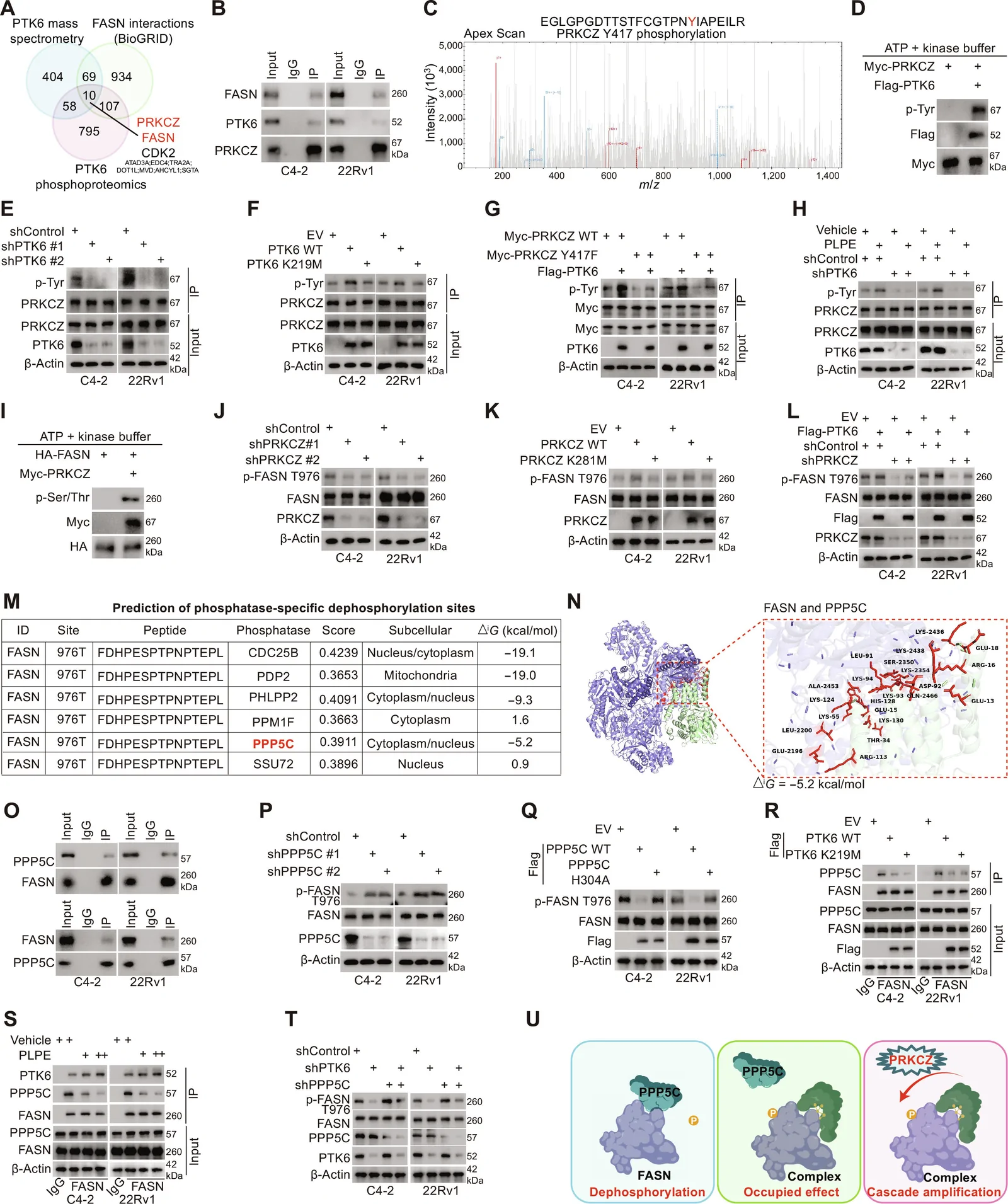
The 1-palmitoyl-2-linoleoyl phosphatidylethanolamine (PLPE)-protein tyrosine kinase 6 (PTK6) complex drives fatty acid synthase (FASN) T976 phosphorylation via a kinase cascade and steric hindrance. (A) Venn diagram of PTK6 protein mass spectrometry of proteins, PTK6 phosphorylation proteomics, and FASN-interacting proteins (BioGRID database). (B) Coimmunoprecipitation (Co-IP) analyses of prostate cancer (PCa) cell lysates were conducted with the indicated antibodies. (C) The peptide information of protein kinase C-zeta (PRKCZ) Y417 in the phosphorylation proteomics of PTK6. (D) A purified PRKCZ substrate and PTK6 kinase were incubated in kinase buffer containing adenosine triphosphate (ATP) for in vitro kinase reactions. Reaction products were analyzed by Western blot for the phosphorylation signal. (E to H) Following transfection with the indicated plasmids or exposure to specific pharmacological agents for 72 h, PCa cells were harvested for Co-IP. (I) A purified FASN substrate and PRKCZ kinase were incubated in kinase buffer containing ATP for in vitro kinase reactions. Reaction products were analyzed by Western blot for the phosphorylation signal. (J to L) Following transfection with the indicated plasmids for 72 h, PCa cells were harvested for Western blotting analysis. (M) Potential phosphatases responsible for the dephosphorylation of FASN at T976 were computationally predicted utilizing the GPSD (https://gpsd.biocuckoo.cn/). (N) The binding energy between protein phosphatase 5 catalytic subunit (PPP5C) and FASN proteins was predicted using AlphaFold 3 and PDBePISA, and the interaction mode was visualized by the PyMOL software. (O) Co-IP analyses of PCa cell lysates were conducted with the indicated antibodies. (P and Q) Following transfection with the indicated plasmids for 72 h, PCa cells were harvested for Western blotting analysis. (R and S) Following transfection with the indicated plasmids or exposure to specific pharmacological agents for 72 h, PCa cells were harvested for Co-IP. (T) Following transfection with the indicated short hairpin RNAs (shRNAs) for 72 h, PCa cells were harvested for Western blotting analysis. (U) Schematic diagram: PPP5C mediates dephosphorylation of FASN at T976 via direct binding. Elevated PLPE facilitates the PTK6–FASN interaction, which induces steric hindrance to hinder PPP5C–FASN binding. Moreover, the PTK6-triggered kinase cascade further amplifies FASN phosphorylation.

Notably, our earlier findings showed that PTK6-driven FASN T976 phosphorylation is not entirely reliant on its intrinsic kinase activity (Fig. 5D) and that the scaffold function of PTK6 partially contributes to enzalutamide resistance in PCa (Fig. 2G). Therefore, we hypothesized that the PLPE-enhanced interaction between PTK6 and FASN might also promote FASN hyperphosphorylation by sterically occluding the binding of specific phosphatases. To test this, we combined predictions from the dedicated GPSD module within the overarching GPS platform, subcellular localization matching, and thermodynamic binding affinity to identify potential candidate phosphatases. Given their similar subcellular localization to FASN and favorable binding affinities, PH domain leucine-rich repeat-containing protein phosphatase 2 (PHLPP2) and protein phosphatase 5 catalytic subunit (PPP5C) were identified as promising phosphatase candidates (Fig. 6M). Notably, Husremović et al. [34] reported that PHLPP2 acts as a pseudophosphatase lacking in vitro catalytic activity. Therefore, we focused our subsequent investigations on PPP5C. Subsequent biochemical and functional analyses confirmed that PPP5C physically interacts with FASN and modulates its phosphorylation level at T976 (Fig. 6N to Q). Moreover, ectopic expression of PTK6 substantially suppressed the interaction between FASN and PPP5C, an effect that was independent of PTK6 kinase activity (Fig. 6R). In parallel, exogenous PLPE supplementation synergistically promoted PTK6–FASN binding while concomitantly disrupting FASN-PPP5C interaction (Fig. 6S). These findings indicate that the PLPE–PTK6 complex competitively prevents FASN-PPP5C binding through steric occupancy, thereby blocking PPP5C-dependent dephosphorylation. Importantly, the down-regulation of FASN phosphorylation caused by PTK6 depletion was only partially reversed by PPP5C knockdown (Fig. 6T), which is consistent with our proposed dual mechanism. Collectively, these findings indicated that the PLPE–PTK6 complex regulates FASN phosphorylation through a dual regulatory mechanism: PRKCZ-mediated kinase activation and PPP5C spatial exclusion (Fig. 6U). This coordinated mechanism drives FASN hyperactivation, instigates cellular lipid metabolic dysregulation, and blunts the therapeutic sensitivity of PCa to enzalutamide.

## Discussion

An age-related decline in basal metabolic rate and ADT-induced metabolic disturbances render metabolic syndrome highly prevalent among patients with advanced PCa. Substantial evidence has established obesity not only as an independent risk factor for multiple malignancies but also as a key driver of resistance to diverse therapeutic modalities, including chemotherapy [35], immunotherapy [36], and targeted therapy [37]. For instance, obesity mediates endocrine therapy resistance in breast cancer via multifaceted mechanisms, such as endocrine dysregulation, chronic inflammation, oxidative stress, and remodeling of the tumor microenvironment [38]. In PCa, Saha et al. [39] also reported that obesity-driven tumor microenvironment remodeling and augmented lipid metabolism represent core mechanisms underlying chemoresistance. Furthermore, excessive intracellular lipid accumulation can sequester lipophilic agents, thereby altering their pharmacokinetic profiles and diminishing their antitumor activity [40]. Consistent with prior reports, our data confirmed that obesity is significantly associated with poor prognosis in patients with CRPC receiving enzalutamide. Notably, a clinical study by Gillessen et al. [41] proposed an “obesity paradox” in patients treated with AR-targeted therapies, wherein obese or overweight patients exhibit unexpectedly better survival outcomes than those with normal BMI. The mechanistic basis for this apparent contradiction remains poorly understood and warrants further investigation in large prospective cohort studies. Emerging lipidomic studies have established that lipids function not merely as structural building blocks of cell membranes but also as critical endogenous signaling molecules that drive tumor progression and therapeutic resistance through multiple mechanisms, including modulation of membrane domain dynamics and activation of oncogenic signaling cascades [42,43]. Using lipidomic profiling, we identified markedly elevated PLPE levels in obese patients treated with enzalutamide. Our results indicate that PLPE acts as a critical molecular bridge linking systemic obesity to intratumoral enzalutamide resistance, thereby offering novel mechanistic insights into obesity-mediated enzalutamide resistance.

The malignant evolution of PCa is propelled by aberrant signaling cascades and the dynamic posttranslational modification of critical regulatory factors [44,45]. PTK6, a cytosolic tyrosine kinase, drives tumor progression by activating multiple oncogenic networks [46]. Because it is devoid of a typical lipid-anchoring SH4 sequence, PTK6 maintains a highly dynamic subcellular distribution [46]. The active pool of PTK6 in PCa cells is primarily localized to the membrane [25,47]. Zheng et al. [47] found that membrane-anchored PTK6 drives the epithelial–mesenchymal transition in PCa. However, the mechanisms regulating PTK6 localization within the cell remain unclear. Notably, PTK6 harbors a distinct lipid-binding domain. This protein–lipid interaction may lead to the accumulation of PTK6 at the membrane and regulate its signaling activity [26]. Our study found that PLPE, which is elevated in tissues from CRPC patients with obesity, plays a critical role in mediating PTK6 membrane localization. By specifically binding to the SH2 domain of PTK6, PLPE initiates the robust membrane recruitment of this kinase. Once the PLPE–PTK6 complex hyperactivates FASN, the ensuing lipid metabolic dysregulation generates excessive palmitic acid. We discovered that this surplus palmitic acid acts as a precursor to synthesize more PLPE and strengthen the membrane localization of PTK6, ultimately forming a positive regulatory feedback loop. This discovery provides new insights into the cellular localization of PTK6 in PCa.

Dysregulated lipid metabolism fuels both the initiation and progression of tumors, especially in PCa, where it affects tumor cell energy supply, signaling, and the tumor microenvironment [48,49]. Abnormal lipid metabolites, particularly saturated fatty acids, drive aggressive disease progression by simultaneously triggering intracellular oncogenic cascades and remodeling the tumor microenvironment [50]. As the central catalytic hub for de novo lipogenesis, FASN fuels tumor progression [51,52], making its targeted inhibition a promising translational avenue for both metabolic and oncological therapies [53,54]. Jin et al. [31] demonstrated that HER2-driven tyrosine phosphorylation of FASN serves as a critical driver in breast cancer progression. This suggests that FASN phosphorylation is critical for its functional regulation. Our study reveals that PLPE serves as an intermediary lipid that facilitates PTK6–FASN interaction, orchestrating FASN activation through a dual regulatory mechanism. Biochemically, the PLPE–PTK6 complex recruits PRKCZ to initiate a kinase cascade, leading to robust FASN T976 phosphorylation. Concurrently, from a structural perspective, PTK6 functions as a noncatalytic scaffold that sterically occludes the access of the phosphatase PPP5C, thereby protecting FASN from dephosphorylation. Accumulating evidence indicates that proteins and posttranslational modifications can act as physical scaffolds to promote, or competitively inhibit, substrate–regulator interactions. This dual regulatory mode reflects signaling cross talk and mechanistic complexity [55,56]. Additionally, Ward et al. [57] demonstrated that endocrine-refractory breast tumors exhibit extensive lipid metabolic reprogramming, and the ensuing lipid buildup antagonizes the antitumor efficacy of FASN inhibitors. Consistent with this phenomenon, our results showed that PLPE interacts with the C-terminal region of FASN, disrupting the binding of FASN inhibitors, thus weakening their therapeutic efficacy. Collectively, these findings offer new perspectives on lipid networks in PCa and provide a theoretical basis for targeting lipid metabolic pathways in clinical treatment.

However, this study has several limitations. The scarcity of human specimens restricted our ultimate sample size, a factor that might temper the broader generalizability and statistical impact of the present data. Longer follow-up duration is required to reliably assess the long-term OS outcomes. Moreover, the long-term prognostic value of intratumoral PLPE levels remains to be further evaluated through large-scale, prospective, and multicenter clinical studies. Furthermore, we were unable to conduct a comprehensive screening of all potential compounds, inhibitors, and candidate molecules. This study focuses on enzalutamide resistance induced by obesity-associated lipid metabolism disorders. Given the complexity of obesity-mediated systemic changes, the PLPE we focused on can only partially mimic such conditions. Other unidentified pathways may contribute to the diminished therapeutic response to enzalutamide treatment. Although we preliminarily demonstrated that PTK6 mediates FASN phosphorylation via a kinase cascade pathway, the underlying downstream effector molecules remain to be further explored. Importantly, the combined application of conventional PTK6 inhibitors merely blocks its kinase function without dismantling its critical scaffolding role. Therefore, the development of PTK6-specific proteolysis-targeting chimeras represents a crucial direction for future therapeutic interventions.

Overall, our findings suggest that obesity-associated PLPE elevation is a key factor contributing to poorer clinical prognosis in CRPC patients undergoing enzalutamide therapy. Nontargeted lipidomics data show increased PLPE levels in tissues from CRPC patients with obesity. Elevated PLPE binds to PTK6, enhances PTK6’s membrane localization, and expands its active kinase pool. Furthermore, PLPE reinforces PTK6–FASN interaction, orchestrating FASN hyperphosphorylation through a dual mechanism: PRKCZ-mediated kinase activation and PPP5C spatial exclusion. The enhanced function of FASN leads to increased palmitic acid, which serves as a substrate for further PLPE synthesis, thereby reinforcing PTK6 membrane retention in a positive feedback loop. The lipid metabolism dysregulation triggered by elevated PLPE in obesity-associated PCa cells forms a vicious cycle, accelerating enzalutamide resistance in PCa. PTK6 inhibitors partially reverse the impact of the PLPE–PTK6 complex on enzalutamide sensitivity. Notably, PLPE binds to FASN and interferes with the binding of FASN inhibitors, thereby attenuating their antitumor efficacy when combined with enzalutamide (Fig. 7).

**Fig. 7.**
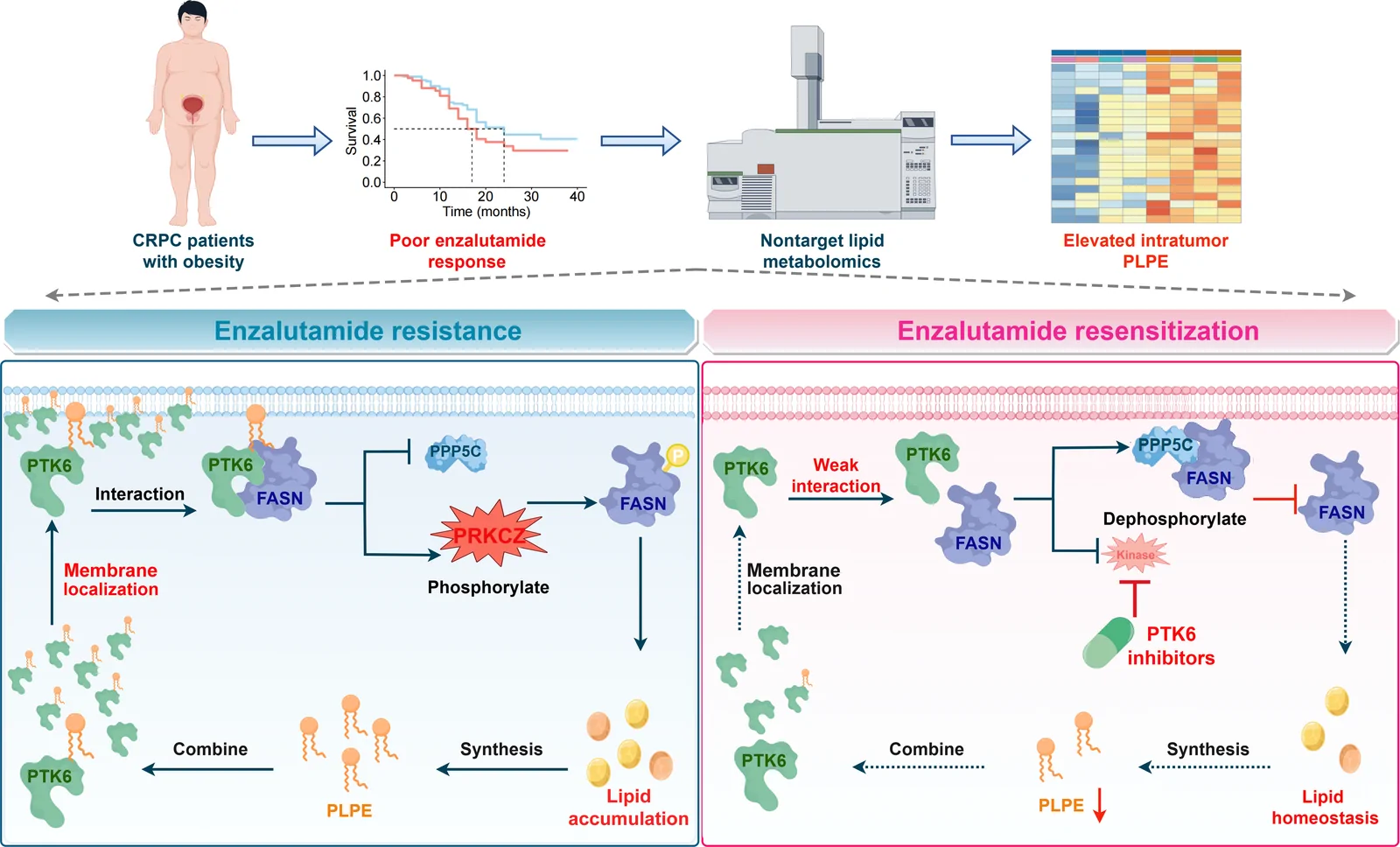
Schematic diagram: 1-palmitoyl-2-linoleoyl phosphatidylethanolamine (PLPE) levels are elevated in tumor tissues from obese castration-resistant prostate cancer (CRPC) patients. Increased PLPE binds to protein tyrosine kinase 6 (PTK6) and enhances PTK6 membrane localization, expanding its active kinase pool. Furthermore, PLPE promotes PTK6–fatty acid synthase (FASN) interaction and activates FASN function, resulting in disrupted lipid metabolism and enzalutamide resistance. PTK6 inhibitors partially reverse the effects of the PLPE–PTK6 complex on enzalutamide sensitivity (created with Figdraw).

## Materials and Methods

### Patient specimens

To investigate the impact of obesity on the sensitivity of CRPC patients to enzalutamide treatment, we performed a multicenter, retrospective cohort analysis in 3 Chinese urological surgery centers (The Second Xiangya Hospital, Qilu Hospital, and Sichuan Provincial People’s Hospital). We retrospectively analyzed the clinical records of 137 CRPC patients who initiated enzalutamide treatment between January 2022 and December 2025. The cohort underwent regular follow-ups, with a median follow-up duration of 20 months. The BMI of all enrolled CRPC patients was calculated based on height and weight measurements obtained at baseline, prior to the initiation of enzalutamide therapy. By the end of the follow-up period, 85 patients experienced PSA progression, 67 patients experienced radiographic progression, and 33 patients had died. Based on the BMI classification criteria established by the National Health Commission of China, patients were stratified into 4 groups: underweight (BMI < 18.5 kg/m^2^), normal weight (18.5 ≤ BMI < 24 kg/m^2^), overweight (24 ≤ BMI < 28 kg/m^2^), and obesity (BMI ≥ 28 kg/m^2^). Clinical outcomes evaluated included PSA-PFS, rPFS, and OS, with the index date defined as treatment initiation. Progression criteria rigorously adhered to the Prostate Cancer Clinical Trials Working Group 3 guidelines and National Comprehensive Cancer Network/European Association of Urology recommendations. Patients without the relevant endpoint event, including those lost to follow-up, were right-censored at their last evaluable assessment for PSA-PFS or rPFS, or at the last date known alive for OS, on or before the data cutoff. The prognostic significance was evaluated through Kaplan–Meier analysis. Furthermore, univariable and multivariable Cox proportional hazards models were utilized to assess independent prognostic values. The experimental protocols involving human subjects were approved by the Medical Ethics Committee of the Second Xiangya Hospital of Central South University (Approval No. LYEC2024-0076). The baseline characteristics of the cohort are provided in Table S1.

### Cell lines

The PCa cell lines 22Rv1 (no. JY185; RRID: CVCL_1045) and C4-2 (no. JY680; RRID: CVCL_4782) were obtained from Jinyuan Biotechnology (Shanghai, China), and the 293T cell line (no. SNL-015; RRID: CVCL_0063) was purchased from SUNNCELL Biotechnology (Wuhan, China). Following short-tandem-repeat-based authentication and the confirmation of mycoplasma-free status via the PlasmoTest detection system (no. rep-pt1; InvivoGen), cells were maintained in a humidified incubator at 37 °C with 5% CO_2_. Specifically, the C4-2 and 22Rv1 lines were grown in RPMI 1640 (Gibco), while 293T cells utilized Dulbecco’s modified Eagle medium (Gibco). All basal media were enriched with 10% fetal bovine serum (no. FBS-CP500; NEWZERUM Ltd.) and a 1% antibiotic cocktail (penicillin–streptomycin; no. BL505A, Biosharp).

For the construction of the enzalutamide-resistant cell line, the parental cells were cultured in RPMI 1640 medium containing enzalutamide (MedChemExpress, no. HY-70002) at a starting concentration of 2.5 μM until reaching approximately 90% confluence. Cells were maintained in medium supplemented with escalating concentrations of enzalutamide, starting from 5 μM and increasing stepwise every 1 to 2 weeks to a final maintenance concentration of 40 μM.

For the generation of genetically modified cell models, cultured cells were preincubated in serum-free Opti-MEM (Gibco, USA) for 12 h postseeding. Subsequent delivery of the indicated knockdown or overexpression constructs (2 μg/ml) was mediated by Lipofectamine 2000 (Invitrogen, USA) as per the vendor’s guidelines. Stable cell lines expressing the indicated plasmids were established through puromycin selection.

### Antibodies and reagents

Primary antibodies were obtained from the indicated suppliers and used at the following dilutions: PTK6 (no. A7497, ABclonal, 1:1,000 dilution), p-PTK6 (phospho Y342) (no. PA5-64567, Invitrogen, 1:1,000 dilution), ATP1A1 (no. 14418-1-AP, RRID AB_2227873, Proteintech, 1:10,000 dilution), cleaved caspase-3 (no. 25128-1-AP, RRID AB_3073913, Proteintech, 1:800 dilution), FASN (no. 66591-1-Ig, RRID AB_2881951, Proteintech, 1:10,000 dilution), p-FASN (phospho T976) (no. TP71286, HuaBio, 1:1,000 dilution), PRKCZ (no. 26899-1-AP, RRID AB_2880675, Proteintech, 1:2,000 dilution), PPP5C (cat. no. 11715-1-AP, RRID AB_2237478, Proteintech, 1:1,000 dilution), p-Tyr (phospho-tyrosine, no. 14-5001-82, RRID AB_2572884, Invitrogen, 1:500 dilution), p-Ser/Thr (phospho-serine/threonine, no. MA5-38234, RRID AB_2898150, Invitrogen, 1:5,000 dilution), β-actin (no. 20536-1-AP, RRID AB_10700003, Proteintech, 1:3,000 dilution), Flag-tag (no. 20543-1-AP, RRID AB_11232216, Proteintech, 1:50,000 dilution), hemagglutinin tag (no. 51064-2-AP, RRID AB_11042321, Proteintech, 1:8,000 dilution), Myc-tag (no. 60003-2-Ig, RRID AB_2734122, Proteintech, 1:10,000 dilution), and glutathione *S*-transferase tag (no. 10000-0-AP, Proteintech, 1:2,000 dilution).

Chemicals and reagents were obtained from the indicated suppliers: enzalutamide (MedChemExpress, cat. no. HY-70002), PLPE (Macklin, cat. no. P969978), biotin-PLPE (Ruixibio, cat. no. R-HFD10), tilfrinib (MedChemExpress, cat. no. HY-110244), TVB2640 (MedChemExpress, cat. no. HY-112829), TVB3664 (MedChemExpress, cat. no. HY-120062), and streptavidin MagBeads (YEASEN, cat. no. 47503ES03). PLPE was dissolved in absolute ethanol (stock at −80 °C). To optimize delivery and stability, working solutions were freshly prepared via conjugation with fatty acid-free bovine serum albumin in culture medium (final ethanol < 0.1%).

### Nontargeted lipidomics analysis

Nontargeted metabolomics and lipidomics were conducted by Applied Protein Technology (Shanghai, China). To ensure high metabolic fidelity, tumor samples were obtained from the viable core with a cold ischemia time of <30 min, immediately snap-frozen, and stored at −80 °C. Only samples with a tumor purity of ≥80% and a necrotic area of <10% (confirmed by hematoxylin and eosin staining) were utilized. Briefly, cellular metabolites and lipids were extracted utilizing a methanol/acetonitrile/water mixture and methyl *tert*-butyl ether, respectively. Subsequent profiling was performed via LC–MS and ultrahigh-performance LC (UHPLC)–MS/MS. During LC–MS/MS analysis, samples were analyzed in a random sequence with interspersed pooled quality control samples to evaluate system stability. Data processing and batch correction were performed using LipidSearch software based on internal standard lipid molecules for data normalization. Raw datasets are available in the China National Center for Bioinformation (CNCB) (No. PRJCA065016: OMIX016873).

### LC–MS/MS quantification

Tissue samples were extracted in 1 ml of methanol via vortexing and ultrasonication. Following centrifugation, the supernatants were filtered (0.22 μm) for analysis. Chromatographic separation was performed on Vanquish Flex UHPLC (Thermo Fisher) utilizing an EXT-C18 column (100 × 2.1 mm, 2.7 μm, 35 °C). The mobile phase (A: aqueous formic acid; B: methanol) was delivered at 0.3 ml/min with the following gradient: 0 to 3 min, 30% B; 5 min, 95% B; 10 to 14 min, 100% B; and 15 to 20 min, 30% B. Detection utilized a TSQ Quantis triple quadrupole mass spectrometer (Thermo Fisher) operating in positive heated electrospray ionization and selected reaction monitoring modes. Key parameters included the following: spray voltage 3.5 kV, sheath/auxiliary gas 50/10 Arb, and ion transfer/vaporizer temperatures of 325/350 °C. Technical assistance was provided by Boyan Labs (Anhui, China).

### RNA sequencing and bioinformatics analysis

Following total RNA extraction, RNA sequencing was conducted by Berry Genomics Technology Co., Ltd. (Beijing, China) utilizing the Illumina HiSeq 2500 sequencing platform (Illumina Inc., San Diego, CA, USA), with 3 biological replicate sets for each experimental group. Raw sequencing reads were subjected to preprocessing procedures to remove ribosomal RNA reads, sequencing adapter sequences, short reads, and other low-quality reads. The resulting clean reads were aligned to the human reference genome (GRCh38/hg38) using the TopHat v2.1.0 software. Missing-value handling was addressed by filtering out genes with zero or extremely low read counts across samples. Data normalization and batch effect correction were concurrently executed within the DESeq2 framework. Differentially expressed genes were identified using the DESeq2 software package, with false discovery rate (FDR) ≤ 0.05 defined as the threshold for statistical significance. Functional enrichment analysis of differentially expressed genes was performed with the clusterProfiler software package. The data generated are publicly available in the CNCB (No. PRJCA065016: subHRA029450).

### Phosphorylation DIA proteomics

Phosphorylation data-independent acquisition (DIA) proteomics analyses were performed by Shanghai BioProfile Biotechnology (China). Cells were lysed, sonicated, centrifuged, and quantified. Proteins were digested by the filter-aided sample preparation method with trypsin (1:50). Peptides were desalted and phosphopeptides were enriched using the High-Select TiO_2_ kit. LC–MS/MS was performed on Orbitrap Astral with Vanquish Neo UHPLC using an 8-min gradient (4% to 99% buffer B, 0.1% formic acid in 80% acetonitrile) in DIA mode. Data were analyzed in Spectronaut 18 (UniProtKB/Swiss-Prot, *Homo sapiens*). Carbamidomethyl C (fixed), oxidation, acetylation, and phosphorylation (variable) were included. FDR < 1%, and site probability ≥ 0.75. Prior to statistical evaluation, missing values were imputed utilizing the *K*-nearest neighbors algorithm, and data normalization was performed based on the median ratio. Differential phosphosites (fold >2 or <0.5, *P* < 0.05) were identified using Perseus and R. The dataset has been uploaded to the CNCB (No. PRJCA065016: OMIX012956).

### Cell proliferation and colony formation assays

Cell viability and IC_50_ values were assessed using the Cell Counting Kit-8 (CCK-8) assay (Beyotime, China). PCa cells in 96-well plates were treated with the indicated compounds for 72 h, followed by incubation with 10 μl of CCK-8 reagent per well for 1 to 2 h at 37 °C. Absorbance was measured at 450 nm. Values from 3 technical replicate wells were averaged to represent a single independent biological replicate.

For colony formation, cells (500 to 1,000 cells/well) were seeded into 6-well plates and cultured for 10 to 14 d. Media were refreshed every 3 d in the continued presence of the indicated compounds to maintain constant pharmacological exposure. At the endpoint, cells were fixed with 4% paraformaldehyde (30 min) and stained with 0.1% crystal violet (30 min). Macroscopic colonies were then photographed and quantified.

### Western blotting and Co-IP

Cells were lysed on ice utilizing radioimmunoprecipitation assay buffer (G2002, Servicebio, China) freshly supplemented with protease and phosphatase inhibitor cocktails (P1005 and P1081, Beyotime, China). Protein concentrations were determined via the bicinchoninic acid assay. Equal quantities of denatured proteins were resolved on sodium dodecyl sulfate–polyacrylamide gel electrophoresis gels and electro-transferred onto 0.45-μm polyvinylidene fluoride membranes (Millipore, USA). Following a 1-h blockade with 5% nonfat milk, membranes were probed overnight at 4 °C with specific primary antibodies. Subsequently, blots were incubated with horseradish peroxidase-conjugated secondary antibodies for 1 h at room temperature. Immunoreactive signals were visualized using an enhanced chemiluminescence substrate (34577, Thermo Fisher Scientific).

For Co-IP and pull-down assays, clarified cell lysates were incubated overnight at 4 °C with target-specific antibodies or biotinylated lipid probes precoupled to magnetic beads. Crucially, isotype IgG or unconjugated blank magnetic beads were utilized as parallel negative controls to strictly eliminate nonspecific binding. Following 6 stringent washes, the enriched complexes were subjected to either immunoblotting validation or protein MS.

### DIA proteomics analysis

Protein LC–MS/MS analyses were conducted by SpecAlly Life Technology (Wuhan, China). Proteins were denatured, reduced, alkylated, and digested with trypsin (1:50). Desalted peptides (via styrene divinylbenzene reversed-phase sulfonate columns) were analyzed utilizing UltiMate 3000 RSLCnano coupled to a Q Exactive HF mass spectrometer (Thermo) operating in DIA mode (one 60K MS1 scan, 350 to 1,250 *m*/*z*; thirty 15K MS2 windows, 28% higher-energy collisional dissociation). Raw data were processed via DIA-NN v1.9.2 (library-free mode) against the human UniProt database. Search settings included ≤2 missed cleavages, fixed carbamidomethylation (C), variable oxidation (M), and N-terminal acetylation, with a strict 1% FDR cutoff. For downstream statistical rigor, protein intensities were normalized utilizing the MaxLFQ algorithm. Missing values were imputed, and batch effects were systematically mitigated. Differentially expressed proteins (fold change >1.5 or <0.67, *P* < 0.05) were identified, with *P* values rigorously adjusted via the Benjamini–Hochberg method for FDR control. The MS datasets have been deposited in the CNCB under accession numbers PRJCA065016 (OMIX017130/OMIX012952).

### Structural modeling and molecular docking

Protein sequences and structures were obtained from UniProtKB and the Research Collaboratory for Structural Bioinformatics Protein Data Bank, and the PLPE structure was retrieved from PubChem. Structures were prepared using Discovery Studio 2019. PLPE–protein docking was performed using AutoDock Vina 1.2.6, with results reported as docking scores (kcal/mol). PTK6–FASN and PPP5C–FASN complexes were modeled using AlphaFold 3, while GRAMM was used for protein–protein docking during construction of the putative PLPE–PTK6–FASN complex. Protein interfaces were analyzed using PDBePISA, with results reported as interface solvation free-energy gains (Δ^i^*G*, kcal/mol). Structures were visualized using PyMOL 3.1.

### Animals

All animal experiments were approved by the Institutional Animal Care and Use Committee of the Second Xiangya Hospital, Central South University (No. 20251344) and conducted in accordance with National Institutes of Health guidelines. Male BALB/c-nu/nu mice (6 to 8 weeks; Boresen Biotech, Changsha, China) were housed under standard specific-pathogen-free conditions. Investigators were blinded to group allocation during tumor assessments. At the study endpoint, tumors were excised and weighed following euthanasia.

### Pharmacological interventions

To ensure optimal pharmacokinetic coverage based on established preclinical evidence, all therapeutics were administered daily (q.d.) via oral gavage. To maximize drug solubility and prolong the in vivo retention time, a highly optimized universal vehicle consisting of 5% dimethyl sulfoxide, 30% PEG300, 5% Tween-80, and 60% double-distilled water was utilized. Enzalutamide was administered at 10 mg/kg/d. The highly selective FASN inhibitors TVB2640 and TVB3664 were administered at 5 and 3 mg/kg/d, respectively. For combination cohorts, agents were co-administered orally.

### Establishment of HFD-induced obesity mouse model

Male mice were randomized into a normal diet (ND; 10% kcal fat, 70% kcal carbohydrates, and 20% kcal protein) or an HFD (60% kcal fat, 20% kcal carbohydrates, and 20% kcal protein) group. Following an 8- to 12-week feeding regimen with weekly monitoring of body weight and food intake, successful establishment of the diet-induced obesity model was confirmed by a ≥20% increase in average body weight relative to age-matched ND controls, corroborated by an elevated BMI.

### Cell-derived xenograft

Following randomization (*n* = 5 per group), preconditioned C4-2 cells (1 × 10^7^ cells/mouse) were subcutaneously inoculated into surgically castrated recipient mice to mimic the androgen-deprived CRPC environment. Tumor dimensions and total body weights were monitored twice weekly, and volumes were estimated utilizing the equation *V* = (length × width^2^)/2. Pharmacological interventions with the indicated therapeutic agents commenced once xenografts reached a palpable volume of approximately 50 mm^3^. Mice were euthanized when tumor volumes exceeded 1,500 mm^3^ or upon meeting predefined humane endpoints (severe tumor ulceration or >20% loss of initial body weight).

### Patient-derived xenografts

Patient-derived xenograft models were established by subcutaneously implanting fresh PCa specimens, surgically obtained from the Second Xiangya Hospital, into surgically castrated NOG mice. The donor patient had confirmed CRPC with continuous progression on prior ADT and maintained an AR-positive, enzalutamide-naïve status. Primary engraftments (F1) were expanded in vivo through serial passaging into secondary recipients (F2). Once F2 tumors reached approximately 300 mm^3^, the tissues were mechanically and enzymatically dissociated and then engrafted into a third cohort of mice (F3 passage) conditioned with the indicated pretreatment regimens. Subsequent evaluations proceeded as previously described.

### Spontaneous PCa model

To induce prostate-specific tumorigenesis via the Sleeping Beauty transposon system, a plasmid cocktail containing pT3-AKT (5 μg), pT3-MYC (5 μg), and pCAG-SB100 (1 μg) in 10 μl of sterile Ringer’s solution was orthotopically microinjected into the prostatic lobes of mice under tribromoethanol anesthesia (250 mg/kg, intraperitoneal) following midline laparotomy. At the study endpoint, mice were euthanized for prostate tissue harvesting and downstream analyses.

### Quantification and statistical analysis

Bioinformatics workflows utilized the R software, whereas standard statistical analyses were performed using GraphPad Prism 8.1. To ensure statistical rigor, technical replicates in quantitative assays were averaged to yield a single value per independent experiment. All quantitative data are uniformly presented as mean ± standard deviation from at least 3 independent biological experiments (*n* ≥ 3).

Statistical differences were assessed via a 2-tailed unpaired Student *t* test for comparisons between 2 individual groups or a one-way analysis of variance followed by Tukey’s post hoc test for multiple group comparisons. Statistical significance was defined as *P* < 0.05, with exact statistical models and biological *n* numbers detailed in the respective figure legends.

## Ethical Approval

The study was conducted in accordance with the principles of the Declaration of Helsinki. It was approved by the Animal Use and Care Committees at the Second Xiangya Hospital, Central South University (No. 20251344). The collection of clinical samples was approved by the Medical Committee of the Second Xiangya Hospital of Central South University (No. LYEC2024-0076). We obtained informed consent from all patients for the use of their specimens.

## Data Availability

Datasets are available from the corresponding authors when reasonably requested. Meanwhile, all data have been deposited at the CNCB (No. PRJCA065016: OMIX012952; OMIX012956; OMIX016873; OMIX017130; subHRA029450).
