# Supplementary material for: Obesity-Induced PLPE–PTK6–FASN Feedback Loop Drives Enzalutamide Resistance by Exacerbating Lipid Metabolic Dysregulation in Castration-Resistant Prostate Cancer
Source: Research (Wash D C). 2026 Oct 15;9:1461. doi: 10.34133/research.1461 (PMC13669505; doi:10.34133/research.1461)
Supplement: Supplementary file 1 [file research.1461.f1.docx]

**Supplementary Materials**

**Supplementary Methods**

**Plasmid construction**

Wild-type and truncated variants of PTK6 and FASN were PCR-amplified from reference cDNAs (Miaolingbio) and inserted into pCDH-3×Flag/HA/Myc backbones. To generate the PTK6-Palm construct, duplexed oligonucleotides encoding the Lyn tyrosine kinase myristoylation/palmitoylation (Palm) sequence (5’-GGCTGCATCAAGAGCAAGAGGAAG-3’) were introduced. Site-directed mutagenesis was executed via fusion PCR. Oligonucleotide sequences for cloning and shRNAs are provided in Table S2.

**Reverse Transcription Quantitative Polymerase Chain Reaction (RT-qPCR)**

Total RNA was extracted using TRIzol Reagent (Invitrogen, Cat#15596026) and quantified using a NanoDrop 2000 spectrophotometer. cDNA synthesis and qPCR were performed using SuperScript IV VILO Master Mix with ezDNase Enzyme (Invitrogen, Cat#11766050) and PowerUp SYBR Green Master Mix (Applied Biosystems, Cat#A25742). Primers were synthesized by BGI Genomics, and their sequences are listed in Table S3.

**Chromatin immunoprecipitation (ChIP)-qPCR analysis**

ChIP assays were executed using Abcam kits for chromatin extraction (#ab117152) and magnetic bead-based IP (#ab156907). Purified targeted DNA enrichment was subsequently quantified via real-time PCR. Locus-specific primer sequences are provided in Table S4.

**Glutathione S-Transferase (GST) Pull-Down Assay**

For GST pull-down assays, pET-GST-expressed proteins from E. coli BL21 (#C600003, Thermo Fisher) were purified using glutathione-agarose beads (#16100, Thermo Fisher). The bead-bound proteins were incubated with cell lysates overnight at 4 °C, extensively washed, and analyzed via SDS-PAGE.

**EdU incorporation assay**

DNA synthesis was evaluated using the BeyoClick™ EdU-555 Kit (C0075S, Beyotime, China). Treated PCa cells were incubated with 10 µM EdU for 2h at 37 °C. Cells were sequentially fixed with 4% paraformaldehyde (15 min), permeabilized with 0.3% Triton X-100 (15 min), and incubated with the Click reaction solution (30 min, dark). Nuclei were counterstained with Hoechst 33342 (10 min). Images were captured using a microscope.

**Apoptosis assay**

Cellular apoptosis was evaluated utilizing the Annexin V-FITC/PI Detection Kit (A211-02, Vazyme, China). Following the indicated treatments, PCa cells were harvested via EDTA-free trypsinization and washed with cold PBS. Cells were resuspended in 1× binding buffer, double-stained with Annexin V-FITC and PI, and incubated for 15 min at room temperature in the dark. Samples were immediately acquired using a flow cytometer. The apoptotic populations were subsequently quantified using FlowJo software.

**Immunofluorescence**

For immunofluorescence, 4% PFA-fixed C4-2 cells were permeabilized, blocked, and incubated with primary antibodies against PTK6 (#A7497, ABclonal; 1:500) and FASN (#66591-1-Ig, Proteintech, 1:1000 dilution). Following secondary antibody incubation and DAPI counterstaining (#TBQ0038, Servicebio), subcellular localization was visualized utilizing a Leica confocal microscope.

**Cellular Thermal Shift Assay (CETSA)**

Cells were treated with vehicle, TVB2640 (5 μM), PLPE (10 μM), or their combination for 24 h at 37 °C. After washing and resuspension, equal numbers of intact cells were aliquoted into PCR tubes and heated at 37, 42, 45, 48, 51, 54, 57, 60, 63, 66, 69, or 72 °C for 3 min, followed by cooling at room temperature for 3 min. Cells were subsequently lysed by repeated freeze–thaw cycles using liquid nitrogen. Lysates were centrifuged at 20,000 × g for 20 min at 4 °C, and equal volumes of the supernatants were analyzed by immunoblotting for FASN. Band intensities were quantified using ImageJ.

**In vitro Kinase Assay**

Kinase and substrate proteins were incubated in kinase reaction buffer supplemented with ATP for 30 min at 30 °C. Reactions were terminated by adding SDS sample buffer and heating. Reaction products were analyzed by SDS-PAGE and immunoblotting using the indicated phospho-specific antibodies.

**Triglyceride (TAG) and Palmitic acid assay**

To precisely quantify specific intracellular lipid contents, cellular triglycerides (TAG) and palmitic acid levels were assessed utilizing a commercial Triglyceride Assay Kit (#BC0625, Solarbio, China) and a Palmitic Acid Assay Kit (COIBO, China), respectively. Briefly, cells were harvested, washed with cold PBS, and subjected to cell lysis. Following centrifugation to remove insoluble debris, the supernatants were collected. All biochemical and enzyme-linked immunosorbent assay (ELISA) procedures were strictly conducted in accordance with the manufacturers' official guidelines.

**Oil Red O (ORO) staining**

For the in situ microscopic visualization of intracellular lipid droplet accumulation, cells cultured in multi-well plates were gently washed with PBS and fixed with 4% paraformaldehyde (PFA) for 30 min at room temperature. The fixed cells were then briefly equilibrated in 60% isopropanol and stained with a freshly filtered 0.5% Oil Red O working solution for 20 min. Following the removal of non-specific background staining via a rapid 60% isopropanol destaining step and subsequent PBS washes, the cell nuclei were counterstained with hematoxylin. Finally, the morphological features of the stained lipid droplets were captured and analyzed utilizing a bright-field inverted light microscope.

**Surface Plasmon Resonance (SPR) assay**

SPR was used to assess the interaction between PLPE and the PTK6 SH2 domain. Recombinant human PTK6 SH2 domain was immobilized on a carboxylated sensor chip by EDC/NHS-mediated amine coupling, followed by blocking of the remaining activated groups. Twofold serial dilutions of PLPE in running buffer (0.625–20 μM) were injected over the sensor surface, and the association and dissociation phases were recorded. Steady-state responses were plotted against PLPE concentration and fitted by nonlinear regression to a one-site binding model, R_eq = R_max × C/(K_D_ + C), where C is the PLPE concentration, R_eq is the equilibrium response, and R_max is the maximal binding response. Both R_max and the equilibrium dissociation constant (K_D_) were estimated from the fit.

**Supplementary Figures**

**Supplementary Figure 1. Obesity is associated with adverse prognosis during enzalutamide treatment in CRPC patients**

A, Kaplan-Meier curves of PSA-PFS, rPFS, and overall survival (OS) across the four newly stratified subgroups: Underweight (BMI < 18.5 kg/m², n = 9), Normal weight (18.5 ≤ BMI < 24 kg/m², n = 86), Overweight (24 ≤ BMI < 28 kg/m², n = 13), and Obesity (BMI ≥ 28 kg/m², n = 29).

B, Kaplan-Meier survival curve of OS comparing the Normal weight and Obesity subgroups, demonstrating a longitudinal trend (P = 0.071).

C, Forest plots illustrating the univariable Cox proportional hazards regression analysis for PSA-PFS and rPFS across standard clinical covariates, continuous BMI, systemic lipidemia, and categorical BMI groups.

D-F, Multivariable Cox regression analyses for PSA-PFS and rPFS evaluating the independent prognostic value of BMI parameterized as a continuous variable (D), the four-category BMI subgroups (E), and Lipidemia (F) in separate multivariable models.

**Supplementary Figure 2. Obesity-associated PLPE elevated in PCa tissue suppresses enzalutamide sensitivity**

A-B, Donut Chart (A) and heatmap (B) of the lipid metabolomic analysis.

C-E, Targeted LC-MS/MS validation of PLPE, including the chromatogram of the authentic PE(16:0/18:2) standard (C), the multi-point calibration curve (D), and representative targeted chromatograms from PCa tumor samples (E).

F, Absolute intratumoral PLPE concentrations in the discovery (N=20) and validation (N=6) cohorts stratified by normal-weight vs. obesity (Student's t-test).

G, Absolute intratumoral PLPE concentrations in the combine cohort stratified by clinical serum lipid status (normal vs. abnormal for lipidemia, triglyceride, and cholesterol) (Student's t-test).

H, Following a 48h incubation with a concentration gradient of PLPE (0 to 1000 μM), the relative cell viability of C4-2 cells was determined via CCK-8 colorimetric analysis (n=3).

I, Time-dependent cell proliferation of C4-2 and 22Rv1 cells exposed to the indicated concentrations of PLPE (5 μM and 10 μM), evaluated via CCK-8 assay (n=3, one-way ANOVA with Tukey's test).

J, Representative fluorescence images and quantification of EdU incorporation in C4-2 cells treated with vehicle, enzalutamide, PLPE, or the combination (n=3, one-way ANOVA with Tukey's test).

K-M, Following randomization, mice were pretreated with either vehicle or PE (16:0/18:2) (1 mg/kg, i.p.) for one week, prior to the subcutaneous inoculation of C4-2 cells to establish the PCa CDX model. The respective vehicle or PLPE treatments were continuously maintained for 5 weeks. Representative tumors images(K), tumor mass (L), and tumor growth curves(M) (n = 3 per group, one-way ANOVA with Tukey's test).

**Supplementary Figure 3. Modeling of obese mice, characterization of metabolic phenotypes, and validation of drug resistance in vivo**

A, Serum levels of total and free testosterone in BALB/c Nude and NOG mice following sham operation or castration (n=5 per group, Student's t-test).

B, Body weight curves of BALB/c Nude mice fed with a normal diet (ND) or a high-fat diet (HFD) for 12 weeks (n=5 per group, two-way ANOVA with Bonferroni's test).

C-E, BALB/c Nude mice were fed with ND or HFD. At the endpoint, samples were collected for the quantification of serum glucose and insulin, serum total cholesterol, free fatty acids, and triglycerides, and tissue PLPE levels (E) (n=5 per group, Student's t-test).

F-K, A subcutaneous CDX model of PCa was established utilizing C4-2 cells in BALB/c Nude mice pre-conditioned with ND/HFD feeding for 12 weeks (F-H) or vehicle/PLPE pretreatment (I-K). Upon the tumors attaining an approximate volume of 50 mm³, mice received vehicle or enzalutamide (10 mg/kg, q.d.) via oral gavage. Representative tumor images (F and I), tumor mass (G and J), and tumor growth curves (H and K) (n = 5 per group, Two/one-way ANOVA with Tukey's test).

**Supplementary Figure 4. The relationship between PTK6 expression and clinical indicators in the PCa cohorts**

A, The information of biotin-labelled PLPE in the mass spectrometry.

B, The binding energy between PLPE and PTK6 proteins was predicted using Autodock vina1.2.6, and the interaction mode was visualized by PyMOL software.

C-H, Box plots showing PTK6 expression stratified by tissue (C), Gleason score (D), N stage (E), T stage (F), Metastasis (G) and Therapy (H).

I-J, Disease-specific (I) and progression-free survival (J) analysis of TCGA-PRAD patients grouped according to PTK6 expression levels.

**Supplementary Figure 5. Non-classical AR pathway enhances transcriptional activation of PTK6**

A, PTK6 transcriptional regulatory network analysis conducted via the ChIP-Atlas database.

B, ChIP-seq profiling of upstream regulators bound to the PTK6 promoter.

C, ChIP-qPCR assessment of AR, TET2, and CXXC5 chromatin association at the PTK6 locus in shRNA-treated enzalutamide-resistant PCa cells (n=3, one-way ANOVA with Tukey's test).

D, Alterations in PTK6 expression following AR knockout were evaluated using the KnockTF 2.0 web server (http://www.licpathway.net:8081/KnockTF/).

E, The relationship between the expression of AR/TET2/CXXC5 and PTK6 in the TCGA-PRAD database was analyzed using the TIMER2.0 web tool (https://compbio.cn/timer2/).

F, Following transfection with the indicated shRNAs for 72h, PCa cells were harvested for Western blotting analysis.

G, The MEXPRESS web tool (https://mexpress.ugent.be/) was utilized to analyze the position of the CpG island in the PTK6 promoter region and the impact of its methylation on PTK6 expression.

H, Schematic diagram: The AR-TET2-CXXC5 complex upregulated the expression of PTK6 in enzalutamide-resistant CRPC cells.

**Supplementary Figure 6. PTK6 is a key molecule that regulates the sensitivity to enzalutamide treatment**

A-F, Following transfection with the indicated plasmids or exposure to specific pharmacological agents for 72h, PCa cells were harvested for Western blotting (A, C and E) or IC_50_ determination (B, D and F).

G, Following transfection with the indicated shRNAs for 72h, PCa cells were further treated with enzalutamide for 24h and subsequently subjected to EdU assays (n=3, one-way ANOVA with Tukey's test).

H-J, Following transfection with the indicated plasmids or exposure to Tilfrinib (2 μM) for 72h, PCa cells were further treated with enzalutamide for 24h. Apoptosis indicators were then evaluated using Western blotting.

**Supplementary Figure 7. PLPE interacts with the PTK6 SH2 domain to promote membrane localization**

(A) Following exposure to the indicated concentrations of PLPE, the relative mRNA levels of PTK6 in PCa cells were determined by RT-qPCR (n=3, one-way ANOVA with Tukey's test).

(B) Following transfection with the indicated shRNAs or exposure to PLPE for 72h, PCa cells were harvested for Western blotting analysis.

(C) Following transfection with a panel of PTK6 variants—wild-type (PTK6 WT), a kinase-dead mutant (PTK6 K219M), a membrane-localization-defective mutant lacking the SH2 domain (PTK6 △SH2), and a constitutively membrane-anchored mutant bearing a palmitoylation sequence (PTK6 Palm, a well-established construct validated in prior literature)—subcellular fractions of PCa cells were extracted and evaluated via Western blotting. WCL: whole cell lysate; CYT: cytoplasm; MEM: biological membrane.

(D) Following transfection with the indicated plasmids for 72h, PCa cells were subsequently cultured with escalating concentrations of enzalutamide for 72h. The IC_50_ of enzalutamide was then quantified with the CCK-8 assay.

(E-F) Whole-cell lysates expressing Flag-PTK6 were incubated with the indicated biotinylated lipid probes or unconjugated biotin, precipitated using streptavidin magnetic beads, and immunoblotted with an anti-Flag antibody.

**Supplementary Figure 8. PLPE regulates the sensitivity to enzalutamide through PTK6**

A-B, Following transfection with the indicated plasmids or exposure to specific pharmacological agents for 72h, PCa cells were subsequently cultured with escalating concentrations of enzalutamide for 72h. The IC_50_ of enzalutamide was then quantified with the CCK-8 assay.

C-D, Following transfection with the specified plasmids or exposure to the indicated chemicals for 72h, PCa cells were harvested for Western blotting analysis.

E-F, Representative tumor images of xenograft models from nude mice under the indicated intervention conditions.

**Supplementary Figure 9. The PLPE-PTK6 complex regulates enzalutamide sensitivity via FASN**

A-G, Following transfection with the indicated plasmids or exposure to specific pharmacological agents for 72h, PCa cells were harvested for IC_50_ determination (A, B and E), Annexin V/PI flow cytometry (C and D), EdU assay (F) and colony formation assay (G) (n=3, one-way ANOVA with Tukey's test).

H-J, Stably transfected C4-2 cells were subcutaneously inoculated into PLPE-pretreated mice. Upon reaching a palpable tumor volume of 50 mm³, mice were treated with enzalutamide (10 mg/kg, q.d.) via oral gavage. Representative tumors images(H), tumor mass (I), and tumor growth curves(J) (n=5 per group, one-way ANOVA with Tukey's test).

**Supplementary Figure 10. The PLPE-PTK6 complex attenuates the antitumor effect of FASN inhibitors combined with enzalutamide**

A, C4-2R cells were co-treated with a fixed concentration of TVB2640 (0.2 μM) or TVB3664 (50 nM) plus increasing concentrations of enzalutamide for 72 h. The IC₅₀ of enzalutamide was then determined via CCK-8 assay.

B, Following exposure to the indicated chemicals for 48h, C4-2 R cells were harvested for Western blotting analysis.

C-G, Following transfection with the indicated plasmids or exposure to specific pharmacological agents for 72h, PCa cells were harvested for IC_50_ determination (C/E), Western blotting (D/F) and EdU assay (G).

**Supplementary Figure 11. Triple-drug combination reverses enzalutamide resistance**

A-D, Following co-treatment with the indicated compound combinations for 72 h, C4-2 cells were harvested for IC₅₀ determination (A and C) and Western blotting analysis (B and D).

**Supplementary Figure 12. PLPE suppresses the anti-tumor effect of FASN inhibitors in PCa**

A, After 48 h of pretreatment with PLPE, PCa cells were further co-treated with escalating concentrations of TVB2640 or TVB3664 in the continued presence of PLPE for 72 h. The IC_50_ of FASN inhibitors was subsequently determined via the CCK-8 assay.

B, Following pretreatment with vehicle or PLPE, C4-2 cells were further cultured with TVB2640 or TVB3664 in the continued presence of PLPE, and subjected to colony formation assays and quantification (n=3, one-way ANOVA with Tukey's test).

**Supplementary Figure 13. FASN T976 phosphorylation promotes its homodimerization**

(A) Following treatment with cycloheximide (CHX) for the indicated times, the protein stability of HA-FASN WT and its phosphorylation mutants (T976E and T976A) in C4-2 cells was evaluated via Western blotting and quantification (n=3).

(B) Representative immunofluorescence images showing the subcellular localization of Flag-FASN WT and its phosphorylation mutants.

(C) Following co-transfection with HA-FASN and Myc-FASN (WT or phosphorylation mutants), PCa cells were harvested for Co-IP assays to evaluate FASN homodimer formation.

**Supplementary Figure 14. PLPE-PTK6 complex promotes lipid synthesis via FASN**

A-G, Following transfection with the indicated plasmids (Flag-PTK6 or shFASN) or exposure to specific compounds (PLPE: 10 μM; TVB2640: 0.2 μM; and TVB3664: 50 nM), C4-2 cells were harvested for cellular TAG or palmitic acid quantification (n=3, one-way ANOVA with Tukey's test).

H-I, Following transfection with the indicated plasmids or exposure to Tilfrinib for 72h, C4-2 cells were harvested for the quantification of endogenous PLPE levels (n=6, one-way ANOVA with Tukey's test).

**Table S1. Baseline characteristics of patients**

| **Characteristic** | **Underweight** | **Normal** | **Overweight** | **Obesity** | ***P* value** |
| --- | --- | --- | --- | --- | --- |
| **N** | 9 | 86 | 13 | 29 |  |
| **Age** | 68.22±11.30 | 67.55±8.63 | 67.77±5.70 | 67.48±7.28 | 0.996 |
| **BMI** | 17.60 (17.40-17.90) | 22.05 (20.90-22.87) | 26.20 (25.60-26.80) | 29.20 (28.50-29.70) | <0.001 |
| **PSA** | 76.20 (21.52-81.44) | 56.51 (18.89-131.86) | 76.31 (15.47-308.40) | 82.10 (27.40-217.60) | 0.559 |
| **ECOG** |  |  |  |  | 0.937 |
| **0** | 6 (66.67%) | 52 (60.47%) | 7 (53.85%) | 18 (62.07%) |  |
| **1** | 3 (33.33%) | 34 (39.53%) | 6 (46.15%) | 11 (37.93%) |  |
| **Gleason score** |  |  |  |  | 0.981 |
| **<8** | 4 (44.44%) | 36 (41.86%) | 5 (38.46%) | 13 (44.83%) |  |
| **≥8** | 5 (55.56%) | 50 (58.14%) | 8 (61.54%) | 16 (55.17%) |  |
| **Type of disease progression at study entry** |  |  |  |  | 0.395 |
| **PSA progression only** | 4 (44.44%) | 50 (58.14%) | 6 (46.15%) | 12 (41.38%) |  |
| **Radiographic progression ± PSA** | 5 (55.56%) | 36 (41.86%) | 7 (53.85%) | 17 (58.62%) |  |
| **Visceral disease (lung or liver)** |  |  |  |  | 0.487 |
| **No** | 7 (77.78%) | 73 (84.88%) | 10 (76.92%) | 21 (72.41%) |  |
| **Yes** | 2 (22.22%) | 13 (15.12%) | 3 (23.08%) | 8 (27.59%) |  |
| **Lipidemia** |  |  |  |  | <0.001 |
| **No** | 7 (77.78%) | 70 (81.40%) | 8 (61.54%) | 11 (37.93%) |  |
| **Yes** | 2 (22.22%) | 16 (18.60%) | 5 (38.46%) | 18 (62.07%) |  |

**Table S2. The shRNA sequences.**

| shRNAs | Forward primer (5′ - 3′) |
| --- | --- |
| shPTK6 #1 | 5'-GATCACCTCTCCCATGACCACAATACTCGAGTATTGTGGTCATGGGAGAGGTTTTTTG-3' |
| shPTK6 #2 | 5'-GATCGTGCAGGAAAGGTTCACAAATCTCGAGATTTGTGAACCTTTCCTGCACTTTTTG-3' |
| shFASN #1 | 5'-GATCCCTACTGGATGCGTTCTTCAACTCGAGTTGAAGAACGCATCCAGTAGGTTTTTG-3' |
| shFASN #2 | 5'-GATCGCTACGACTACGGCCCTCATTCTCGAGAATGAGGGCCGTAGTCGTAGCTTTTTG-3' |
| shAR #1 | 5'-GATCGTAATAGTGGTTACCATTCATCTCGAGATGAATGGTAACCACTATTACTTTTTG-3' |
| shCXXC5 #1 | 5'-GATCCAACAGAAGAAAGGGCTTCTTCTCGAGAAGAAGCCCTTTCTTCTGTTGTTTTTTG-3' |
| shTET2 #1 | 5'-GATCAGTGTTCCGCAATTTACATCTCGAGATGTAAATTGCGGAACACTTTTTTG-3' |
| shPRKCZ #1 | 5'-GATCCGCGTGATTGACCCTTTAACTCTCGAGAGTTAAAGGGTCAATCACGCGTTTTTTG-3' |
| shPRKCZ #2 | 5'-GATCCTGGTGCGGTTGAAGAAGAATCTCGAGATTCTTCTTCAACCGCACCAGTTTTTTG-3' |
| shPPP5C #1 | 5'-GATCCCAGATCACTTTCACCTCCTTCTCGAGAAGGAGGTGAAAGTGATCTGGTTTTTTG-3' |
| shPPP5C #2 | 5'-GATCCCACGAGACAGACAACATGAACTCGAGTTCATGTTGTCTGTCTCGTGGTTTTTTG-3' |

**Table S3. The primer sequence for RT-qPCR.**

| Gene  (Human） | Forward primer (5′ - 3′) | Reverse primer (5′ - 3′) |
| --- | --- | --- |
| β-actin | 5'-GCGTGACATTAAGGAGAAG-3' | 5'-GAAGGAAGGCTGGAAGAG-3' |
| PTK6 | 5'-CCAAGTATGTGGGCCTCTGG-3' | 5'- CCAAAGAACCACGGTTCCGA-3' |

**Table S4. The primer of PTK6 for ChIP-qPCR**

| Gene (Human) | Forward primer (5′ - 3′) | Reverse primer (5′ - 3′) |
| --- | --- | --- |
| Primer 1 | TGAGCTTCCAGTTCTGGCAG | GCACCAAACCTGTTCAGCTG |
| Primer 2 | CCACCCCTTGCAGACTTTGA | CTTCTCCCGACTCATGCCTG |
| Primer 3 | CACTCCCTCTTCCCTGCTTG | CTTCCCAACCCTCAGAGCTG |
